# Characterization of the *Esi3/RCI2/PMP3* gene family in the Triticeae

**DOI:** 10.1186/s12864-018-5311-8

**Published:** 2018-12-11

**Authors:** Sabrina C. Brunetti, Michelle K. M. Arseneault, Patrick J. Gulick

**Affiliations:** Biology Department, Concordia University, 7141, Sherbrooke, W. Montreal (Quebec) H4B 1R6 Canada

**Keywords:** Early salt induced gene family, *Esi3*, Tissue-specific expression, *Rare Cold Inducible 2*, *RCI2*, *Plasma Membrane Protein 3*, *PMP3*, RNA-seq

## Abstract

**Background:**

Members of the *Early Salt Induced 3* (*Esi3/RCI2/PMP3*) gene family in plants have been shown to be induced in response to both biotic and abiotic stresses and to enhance stress tolerance in both transgenic plants and *Saccharomyces cerevisiae*. *Esi3* was first identified as a salt stress induced gene in the salt tolerant wild wheat grass, *Lophopyrum elongatum*, and subsequently homologous genes in many other species were found to be members of the gene family. These include *Arabidopsis thaliana* and *Oryza sativa* where they are referred to as *Rare Cold Inducible 2* (*RCI2*), and *Zea mays* where they are referred to as *Plasma Membrane Protein 3* (*PMP3*)*.* This study characterizes the *Esi3* family members in *Triticum aestivum* and explores the tissue specific expression patterns of the gene family members as well as their response to a variety of environmental stresses.

**Results:**

The *Esi3* gene family was found to have a total of 29 family members comprised of ten paralogous groups in the hexaploid *T. aestivum*. Each paralogous group contains three homeologous copies, one in each of the A, B and D genomes with the exception of *Esi3–2* which is missing the B copy. The genes of the *Esi3* gene family were also identified in four other monocot species, *Aegilops tauschii*, *Hordeum vulgare*, *Secale cereale* and *Sorghum bicolor*, and were confirmed or corrected for *Brachypodium distachyon, Oryza sativa* and *Zea mays,* as well as the dicot *Arabidopsis thaliana*. Gene expression of the *Esi3*s was analyzed using tissue-specific, abiotic and biotic stress RNA-Seq 454 sequence libraries and Affymetrix microarray data for *T. aestivum*.

**Conclusions:**

Members of nearly all paralogous groups of the *Esi3* genes in *T. aestivum* have altered gene expression in response to abiotic or biotic stress conditions. In addition, there are modest differences in gene expression among homeologous members of the gene family. This suggests that the *Esi3* gene family plays an important role in the plants response to the stresses presented in this study. The *Esi3–9* in *T. aestivum* has a unique N terminal extension placing it into Group III, a new group for the *Esi3/RCI2/PMP3* gene family.

**Electronic supplementary material:**

The online version of this article (10.1186/s12864-018-5311-8) contains supplementary material, which is available to authorized users.

## Background

Members of the *Esi3/RCI2/PMP3* gene family have been shown to be up-regulated by environmental stresses and to contribute to stress tolerance in studies using model species. The *Early Salt-Induced 3* (*Esi3*) gene was initially identified as a salt-stress induced gene in *Lophopyrum elongatum*, a salt tolerant and close relative of bread wheat, *Triticum aestivum* [1]. It was reported to be more strongly induced by stress in *L. elongatum* than the less tolerant *T. aestivum* and the intermediately salt tolerant amphiploid derived from a cross between *L. elongatum* and *T. aestivum* [2]. A close homolog was also identified as a low temperature induced gene in *Hordeum vulgare* (barley), *Blt101* [3]. The genes encode small molecular weight hydrophobic proteins that are members of gene families that have been identified in a wide range of eukaryotic and prokaryotic organisms including more than 150 plant species [4]. The homologous gene family in *Arabidopsis thaliana*, referred to as *Rare Cold Inducible 2* (*RCI2*) [5], has been shown to be up-regulated by environmental stresses such as low temperature, salt and dehydration [6–8]. The eight gene family members in *Arabidopsis* have been divided into two groups; Group I, which encode proteins of 52 to 64 amino acids, and Group II which encode longer proteins, of approximately 60 to 89 amino acids, with longer C terminal hydrophobic extensions which contain charged amino acids [4]. A homologous gene has also been described in *Saccharomyces cerevisiae*, known as *Plasma Membrane Protein 3* (*PMP3*), the deletion of which resulted in a decrease in plasma membrane potential and caused sensitivity to Na^+^ and cytotoxic cations such as tetramethylammonium and hygromycin B. This suggests that this protein contributes to the regulation of intercellular ion homeostasis by controlling plasma membrane potential and preventing excessive Na^+^ influx [7]. The Arabidopsis *RCI2A* gene was shown to complement the deletion of yeast *PMP3*, indicating that they are functionally interchangeable [7–9]. The *Δpmp3* mutation in *S. cerevisiae* can be complemented by many but not all members of the gene families from other plant species including *Zea mays* [10], *Medicago truncatula* [11], *Oryza sativa* [8] and the alkali grass, *Puccinellia tenuiflora* [12].

Overexpression of *RCI2A* in transgenic Arabidopsis plants was shown to decrease Na^+^ uptake, mitigate the salt-induced damage and enhance growth under salt stress conditions [13], whereas the disruption of *RCI2A* led to over-accumulation of Na^+^ and increased salt sensitivity [14] which suggests that RCI2 proteins play major roles in ion homeostasis in response to salt stress. These results were confirmed by experiments in which the *MpRCI2* from plantain, *Musa paradisiaca*, was overexpressed in Arabidopsis [15]. Overexpression of *RCI2* in *Nicotiana tobacum* increased tolerance to low temperatures [16]. The overexpression of the *Z. mays* gene *ZmPMP3–1* in the *A. thaliana rci2* mutant background resulted in higher levels of salt tolerance than either the *rci2* mutant or wild type (WT) plants [10]. In response to NaCl treatment, the transgenic plants showed lower levels of Na^+^ accumulation and enhanced K^+^ accumulation relative to the mutant or the WT, though the increased K^+^ accumulation phenotype was not observed in lines that overexpressed *AtRCI2A* [13].

While the mechanisms by which these proteins act are not well known, seven of the *A. thaliana RCI2* proteins [4], eight *RCI2*-like proteins identified in *Zea mays* [10] as well as several *RCI2*-like genes from *M. truncatula* [11] have been shown to localize to the plasma membrane when they are transiently expressed as green fluorescent protein (GFP) fusion proteins in onion epidermal cells. The Arabidopsis and *M. truncatula* studies also reported single members of the gene family that were also localized to internal cellular membranes [4, 11]. The Esi3/RCI2/PMP3 proteins are not thought to directly affect sodium transporters; combining the *Δpmp3* deletion with the deletion of the two major Na^+^ expelling transporters in *S. cerevisiae*, *Δpmr2* and *Δnha1*, increased salt sensitivity of the mutants which suggests that PMP3 does not likely act directly in conjunction with those ion pumps. The *Esi3/RCI2/PMP3*-encoded proteins are predicted to have two transmembrane domains which alone would not be sufficient to form transmembrane pores, though oligomeric complexes have been hypothesized to have a potential to do so [5]. Recent studies carried out with PMP3 in Saccharomyces suggest that it plays a role in plasma membrane organization and is part of the regulatory mechanism for vesicle movement between the plasma membrane and vacuole [17]. Additionally, it is involved in the regulation of levels of phosphoinositides and sphingolipids [17].

Members of the *Esi3/RCI2/PMP3* gene families studied in *A. thaliana* and *Z. mays* displayed differential expression in developing tissues and in response to various abiotic stresses, which suggests that they are involved in separate signaling pathways and that those gene family members may play divergent roles [5, 10, 18]. The *Esi3*s that were first identified in the highly salt tolerant *L. elongatum* are thought to be members of a small gene family in the genomes of the closely related Triticeae species and to have gene family members represented in the three genomes of hexaploid bread wheat, *T. aestivum*. The allohexaploid genome of *T. aestivum* is the result of two polyploidization events. The first event occurred between the diploid *Triticum urartu* and a species thought to be closely related to *Aegilops speltoides*, which donated the A and B genomes respectively, and which resulted in the tetraploid species *Triticum turgidum*. The second allopolyploid speciation event involved a cross between *T. turgidum* and *Aegilops tauschii*, which contributed the D genome and occurred approximately 8000 years ago [19–21]. The three genomes have a high degree of synteny and DNA sequence similarity.

Here we report the identification of all homologous sequences of *Esi3* genes within the genome of *T. aestivum*, and analyze the differential expression patterns of these genes. *Esi3* gene family members were identified in cDNA and genomic databases. In addition, gene expression levels in response to stress treatments were characterized from microarray and transcriptome sequence sources. To elucidate the evolutionary relationships between the *Esi3* homologues across plant species, the gene sequence similarity was compared to those of other monocot species including *Ae. tauschii*, *Sorghum bicolor*, *Brachypodium distachyon*, *Hordeum vulgare*, *Secale cereale*, *O. sativa*, *Z. mays*, and to the dicot *A. thaliana*.

## Results

### *Esi3* genes in *Triticum aestivum*

Twenty-nine *Esi3/RCI2/PMP3* genes were found in the hexaploid genome of *T. aestivum*. These represent ten paralogous loci in the haploid genome, each with three homeologous copies in the A, B and D genomes with the exception of *Esi3–2* for which the B copy was not identified (Table 1). In most cases, the sequences of the gene family members were confirmed in three independent sequence databases, the International Wheat Genome Sequencing Consortium (IWGSC) database of genomic chromosomal survey sequences [22], and the transcriptome shotgun assembly (TSA) and expressed sequence tag (EST) databases for *T. aestivum* at the National Center for Biological Information (NCBI). Additional confirmation of genome assignment and delineation of coding sequences (CDS) were obtained from the databases for other species of the Triticeae tribe. There was no evidence for an *Esi3–2-B* gene copy in the B genome of *T. aestivum* found in the IWGSC genomic sequence database, the EST or TSA databases at NCBI nor in the databases for other B genome-containing species, *T. turgidum* and *Aegilops speltoides.* All members of the gene family had two exons and one intron. Annotations for twenty-four of these gene family members agreed with the most current annotation of the *T. aestivum* genome assembly at the Ensembl Plants database [23] with the exception of five genes (*Esi3–6-B* and -*D*, *7-B*, *10-A* and *10-D*). The genes that did not agree with our annotations have either extended N terminal domains, mismatches in the protein sequence, or a lack of annotations as protein coding regions; these differences are listed in Additional file 1: Table S1.

**Table 1:** *Esi3* genes of *Triticum aestivum*

Gene	Genome	EST identifier	TSA identifier	Chromosome	Start Codon^b^	Alignment	Exon 1^a^	Intron	Exon 2^a^	a.a^c^
*Esi3–1*	A	CJ595172	JP881209.1	4AS	122,736,187	+/−	83	98	82	54
	B	JZ888897.1	JP881208	4BL	424,862,167	+/+	81	98	84	54
	D	CJ697409.1	JP881207.1	4DL	343,061,456	+/+	81	95	84	54
*Esi3–2*	A	CD881671.1	HAAB01071723.1	4AS	122,627,489	+/−	81	99	84	54
	D	BU099288.1	HAAB01071724.1	4DL	343,088,889	+/+	81	99	84	54
*Esi3–3*	A	CV767975.1	HP633215.1	5AL	561,693,119	+/+	88	140	134	73
	B	CJ925039.1	JP824371.1	5BL	541,736,786	+/+	88	110	134	73
	D	CJ595273.1	GFFI01135300.1	5DL	444,852,365	+/+	90	119	132	73
*Esi3–4*	A	CA665474.1	ND	1AS	65,759,553	+/−	84	161	84	55
	B	CJ901293.1	JV986506.1	1BS	108,210,663	+/−	84	162	84	55
	D	CJ685142.1	JV989019.1	1DS	66,987,844	+/−	84	191	84	55
*Esi3–5*	A	BE497086.1	HAAB01033003.1	2AS	100,129,674	+/−	90	93	84	57
	B	CN010305.1	JV846264.1	2BS	152,175,965	+/−	90	105	84	57
	D	CJ854183.1	HP629889.1	2DS	100,318,450	+/−	90	140	84	57
*Esi3–6*	A	CJ671046.1	HAAB01051397.1	7AL	692,509,547	+/−	93	110	138	76
	B	CJ562290.1	HAAB01051396.1	7BL	679,884,026	+/−	96	136	138	77
	D	CJ559253.1	HAAB01051398.1	7DL	600,502,457	+/−	96	123	138	77
*Esi3–7*	A	BJ261574.1	GFFI01140352.1	7AS	102,537,985	+/−	96	109	135	76
	B	CJ725702.1	GFFI01052110.1	7BS	55,853,830	+/−	99	96	144	79
	D	CJ825516.1	GFFI01131578.1	7DS	100,281,019	+/−	96	96	132	76
*Esi3–8*	A	CD909025.1	ND	1AS	42,505,283	+/−	81	95	84	54
	B	ND	HAAB01084472.1	1BS	62,681,663	+/+	81	120	84	54
	D	CJ648786.1	HAAB01084471.1	1DS	42,845,718	+/−	81	115	84	54
*Esi3–9*	A	ND	HAAB01083453.1	2AL	775,064,923	+/+	240	170	84	107
	B	BJ243843	ND	2BL	785,821,555	+/−	318	171	84	133
	D	BJ243706.1	GAEF01014403.1	2DL	649,781,640	+/−	276	170	84	119
*Esi3–10*	A	HX161660.1	GFFI01106676.1	5AL	561,688,908	+/+	90	141	126	71
	B	CA611646.1	HAAB01089382.1	5BL	541,683,185	+/+	90	1111	126	71
	D	BJ278420.1	HAAB01084536.1	5DL	444,743,823	+/+	90	984	126	71

Notes: *ND* not determined/not detected. GenBank EST and TSA identifiers are representative. The database has multiple matches for most *Esi3*s

^a^Exon lengths are the CDS, the UTRs are not included

*Esi3–9-B* has a non-consensus splice site sequence. The *Esi3–9-D and Esi3–10-A* EST has partial coverage

^b^Position (bp) of start codon (ATG) on pseudomolecules on IWGSC RefSeq v1.0 database

^c^ a.a - amino acid

All members of the *Esi3* gene family have a core conserved protein sequence encoding two transmembrane domains. The gene family can be delineated into three groups based on the presence or absence of C terminal or N terminal extensions of the CDS. Group I includes five of the paralogous gene sets*, Esi3–1, −2, −4, −5,* and *−8*, which encode proteins of the classical *Esi3/RCI2/PMP3* lengths of 54–57 amino acids with two conserved transmembrane domains. Group II contains four paralogous sets of genes, *Esi3–3, −6, −7*, and *−10* which encode proteins between 71 to 79 amino acids in length that include C terminal extensions between 14 and 20 amino acids similar to the Group II genes described in Arabidopsis and other species [5]. These extensions are rich in hydrophobic amino acids but also contain charged amino acids. Group III contains *Esi3–9-A, -B* and -*D* genes that encode N terminal extensions of 49–79 amino acids relative to the other members of the gene family. These N terminal extensions were rich in hydrophobic amino acids and have charged amino acids distributed throughout, a pattern that is similar to the C terminal extensions seen in *Esi3–3*, *−6*, *−7*, and *−10*. At the junction between the N terminal extensions and the conserved domains the proteins had runs of five consecutive valines followed by five prolines. Esi3–9-like proteins with long N terminal extensions similar to those described above are not found in Arabidopsis nor in the *Esi3/RCI2/PMP3* gene families in other well characterized species, and so we have designated them as Group III.

The degree of nucleotide sequence similarity for paralogous copies of the *Esi3* genes of *T. aestivum* within the core conserved sequences, excluding the N or C terminal extensions, for paralogous members of the gene family ranged from 62 to 95% (Additional file 2: Table S2). Amino acid sequences among paralogous gene copies ranged between 47 to 93% identity. The three homeologous gene copies for each of the paralogous groups had amino acid sequence identity ranging from 95 to 100%. The Group I genes, *Esi3–1, −2, −4, −5,* and *−8*, had 100% amino acid sequence identity among homeologous copies (Additional file 2: Table S2). Homeologous copies of *Esi3* genes were localized to homeologous chromosomes. The ten paralogous *Esi3* genes were localized to five different chromosomes. Two pairs of genes appear to be in tandem arrangement. The two most similar pairs of paralogs, *Esi3–1* and *Esi3–2*, which share approximately 92% nucleic acid identity, were both localized to chromosome 4. The two genes are approximately 109 kb and 27 kb apart on the short arm of chromosome 4A and the long arm of chromosome 4D, respectively. *Esi3–3* and *Esi3–10* which are approximately 90% identical are roughly 4 kb, 54 kb and 109 kb apart on chromosomes 5A, 5B and 5D, respectively [22]. In the *Ae. tauschii* genome assembly *Esi3–1* and *Esi3–2* are 20 kb apart on chromosome 4, and *Esi3–3* and *Esi3–10* are approximately 57 kb apart on chromosome 5 [24, 25].

Genes with high sequence similarity to the *Esi3/RCI2/PMP3* gene family members are found in a wide range of plant species including monocots, dicots, and mosses. *Esi3–9-*like genes, which have long N terminal extensions with high sequence similarity, were only identified in other species in the Triticeae. Searches in the GenBank protein databases identified homologous gene family members with similar N termini in *Ae. tauschii, H. vulgare* and *Secale cereale* (XP_020160007.1, BAK07288.1, GCJW01018134.1, respectively; Additional file 3: Table S3) though an *Esi3*-like gene with a long N terminal extension but with low sequence similarity in this domain was identified in the salt tolerant dicot *Eutrema salsugineum* (formerly *Thellungiella halophile,* GBKH01000241.1). *Esi3*s encoding proteins with C terminal extensions, like those of *Esi3–3, −6, −7* and *−10* were found in the EST databases for many other monocotyledonous and dicotyledonous species but not in the moss, *Physcomitrella patens*.

*Esi3/RCI2/PMP3* gene family members were also identified in other monocotyledonous species in which the complete *Esi3/RCI2/PMP3* gene families were not previously described. Using the nucleotide collection, EST and TSA databases at GenBank, ten *Esi3* gene family members were identified in two other species of the Triticeae: *Secale cereale* and *Ae. tauschii*. The gene family in *H. vulgare* is comprised of nine members and includes two genes that had previously been reported [3, 26]. The complete *Esi3* gene family composition is summarized in Additional file 3: Table S3. Seven *Esi3/RCI2/PMP3* gene family members were also identified in *Sorghum bicolor*, one of which was reported by Khurana et al., 2015 [26] (Additional file 3: Table S3). Two novel *Esi3*/*RCI2/PMP3* gene family members were identified in *B. distachyon* in addition to the six gene family members previously described [5]. A small number of discrepancies were found between the previously described *Esi3/RCI2/PMP3* gene families for *O. sativa* and *Z. mays* and the Ensembl Plants database. There were also a number of differences between our compiled amino acid sequences and the annotation of the genes in BLAST at Ensembl Plants [23]; these are summarized in Additional file 3: Table S3 and Additional file 1: Table S1. Protein sequences for the *Esi3/RCI2/PMP3* gene families for these species can be found in Additional files 4 and 5: Sequences S4 and S5.

### Tissue-specific expression of *Esi3* genes

**Fig. 1:**
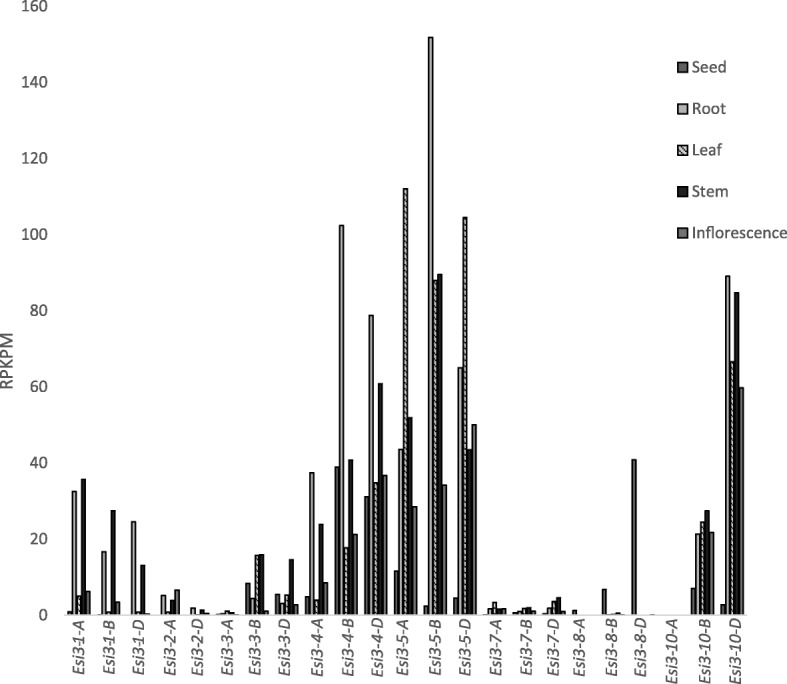
Tissue-specific gene expression. *Esi3* gene expression in tissues of *T. aestivum*, the tissues analyzed included the seed at the whole plant ripening stage, the root at the cotyledon emergence stage, the leaf when the seed was 30 to 50% developed, the stem at the two nodes or internodes visible stage, and the inflorescence when maximum stem length was reached. *Esi3–6* and *Esi3–9* are not graphed because the RPKPM values were lower than 1.0 across all tissue types for all homeologous copies. Quantification is from RNA-seq data and values were normalized to reads per kilobase per million (RPKPM)

The members of the *Esi3* gene family showed varied tissue-specific patterns of gene expression in both transcriptome and microarray datasets. One transcriptomic analysis of five tissues including developing seed, root, leaf, stem and inflorescence [27] was assayed for the 29 gene family members; expression levels ranged from 151.8 RPKPM to undetectable (Fig. 1 and Additional file 6: Table S4). Homeologous groups within *Esi3–1, −2, −3, −4*, *−5* and *−10* had relatively high level of expression compared to *Esi3–6, −7, −8,* and *−9*. Most paralogous groups that were highly expressed had high levels of expression in more than one tissue type. For example, *Esi3–5*, the most highly expressed paralogous group, was highly expressed in the root, leaf and stem. *Esi3–5* was also expressed in the seed and inflorescence but at lower levels. In tissues in which *Esi3* gene expression was detected, all three members of the homeologous groups were detected, though at different levels, with the exception of *Esi3–10-A*. The genome of origin of the most highly expressed homeolog varied from among the paralogs, for example for both *Esi3–1* and *Esi3–2* the A homeolog was more highly expressed than the B or D copy, whereas for *Esi3–10* the D homeolog was detected at higher levels than its corresponding homeologs in all tissues with the exception of the seed (Fig. 1). The extensive transcriptome analysis of seventy-one tissue types in Azhurnaya Spring Wheat also showed that each paralog has varying expression across the tissue panel (Additional file 7: Table S5) [28]. Anther tissue showed relatively high levels of expression of *Esi3–1-A, Esi3–4-A, -B, −D, Esi3–5-A, −D* and *Esi3–9-A, -B* (Additional file 7: Table S5). *Esi3–8-A* had high levels of expression in different tissues of grains at various stages of development but was undetected in most other tissue types. *Esi3–6* had low levels of expression across all tissue types assayed (Additional file 7: Table S5). *Esi3–5-A, -B* and *-D* had high expression levels in root tissues at different stages of development with the highest levels in the root at the seedling stage (Additional file 7: Table S5). This extensive analysis indicates that the *Esi3* gene family members have very dynamic tissue specific and developmentally regulated patterns of expression.

Tissue-specific expression was also analyzed in a panel of thirteen tissue types (Additional file 8: Figure S1 and Additional file 9: Table S6) assayed by the Affymetrix 61 k wheat microarray dataset described by Schreiber et al., 2009 [29]. All paralogous gene family groups were represented on the microarray with the exception of *Esi3–6*. We note that the microarray cannot distinguish between homeologous gene copies in wheat and expression levels were taken as those of homeologous groups that include the A, B and D copy of each paralogous group. The most striking result from the microarray data was the high level of expression of *Esi3–9* in the anthers and its low levels of detection in any other tissue (Additional file 8: Figure S1 and Additional file 9: Table S6). Gene family members *Esi3–1* to *Esi3–5* have relatively high levels of expression in all tissues, a finding that was similar to the RNA-seq data noted above. In addition low levels of expression of *Esi3–1*, *−8* and *−10* were detected in the anthers, and there were low levels of expression of *Esi3–2, −3* and *−9* in the immature inflorescence*. Esi3–3* showed low levels of expression in the caryopsis 3–5 days after pollination. *Esi3–7* and *Esi3–10,* overall have very low levels of expression across all tissue types assayed.

### Expression of *Esi3* genes under drought and temperature stress conditions

**Fig. 2:**
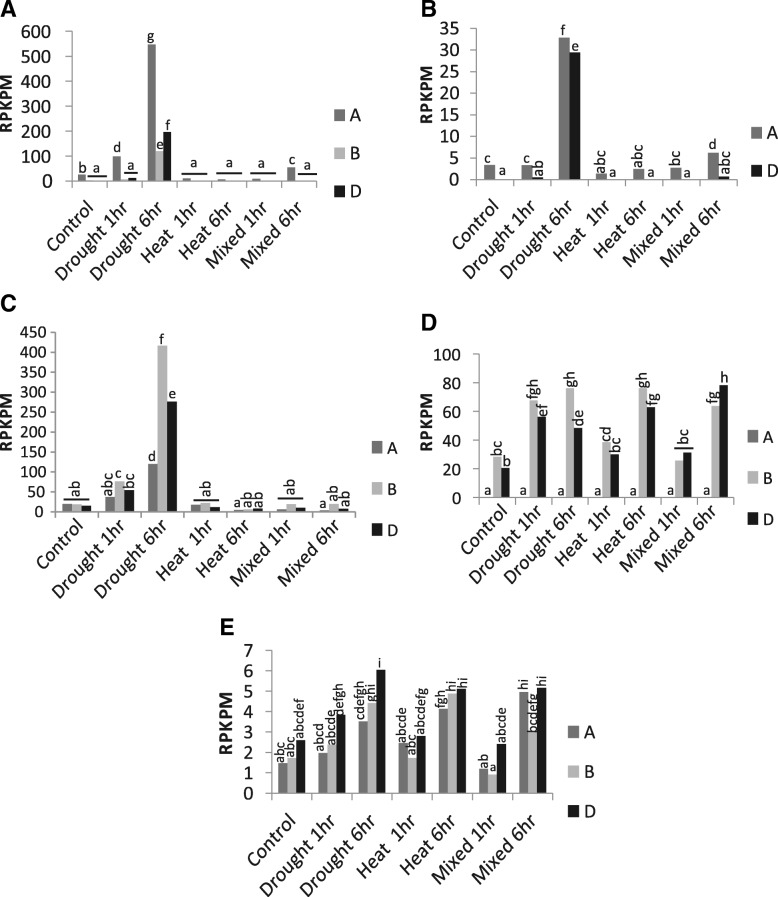
Short term osmotic and heat stress. *Esi3* gene expression in seedling leaves in response to drought, heat, and combined drought and heat at 1-h and 6-h treatment times. Measurements are based on RNA-seq data. Gene expression differences for **a** *Esi3–1*, **b** *Esi3–2*, **c** *Esi3–3*, **d** *Esi3–4*, and **e** *Esi3–7* are represented in reads per kilobase per million (RPKPM). *Esi3s* with significant expression changes are shown. Letters signify Duncan’s multiple range values

The *Esi3* gene family’s expression in leaf tissue in response to osmotic stress and high temperature identified five homeologous groups of the gene family that had significant changes in expression (Fig. 2 and Additional file 10: Table S7). Transcriptomic data from Liu et al., 2015 [30] characterized global changes in gene expression in response to short term osmotic stress treatment with PEG and high temperature treatments in seedlings grown on petri dishes. The experiments revealed a strong induction of gene expression for members of the homeologous groups *Esi3–1* and *−3*, and modest induction of *Esi3–2*, *−4,* and *−7*. All three homeologs of *Esi3–1* were up-regulated more than two fold by one hour of osmotic stress and up-regulated over 20 fold by six hours of stress. The *Esi3–1-A* copy had the highest level of expression in control conditions and was induced 20 fold with six hours of osmotic stress, whereas the B and D copies of the gene had much lower levels of expression in the control plants but were induced 44 and 89 fold, respectively. Nevertheless, the *Esi3–1-A* copy had the highest absolute level of expression in the stressed plants. The *Esi3–1* homeologs were down-regulated by heat treatment and by combined osmotic stress and heat treatment of one hour, however the A copy of the gene was induced two fold by six hours of the combined heat and osmotic stress treatment (Fig. 2 and Additional file 10: Table S7). Similarly, the *Esi3–3-B* copy was highly up-regulated under six hours of osmotic stress. All homeologs of *Esi3–5* had relatively high levels of expression in control conditions, and were down-regulated by drought, up-regulated by heat treatment and up-regulated by combined drought and heat treatment (Additional file 10: Table S7)*.* Alternatively, all homeologs of *Esi3–2* were highly induced after 6 h of drought meanwhile the homeologs are unaffected by the other stress conditions (Fig. 2). *Esi3–10-D* was up-regulated 5 fold after six hours of mixed heat and drought stress, compared to the 0.5–2-fold induction under the other conditions (Additional file 10: Table S7). *Esi3–4* and *Esi3–7* were moderately up-regulated by drought, heat and combined drought and heat treatment (Fig. 2). Most of the homeologous members of the *Esi3* gene family responded similarly to stress conditions, though the absolute level of expression and the degree of change in response to stress varied a few fold among the members of the homeologous groups.

**Fig. 3:**
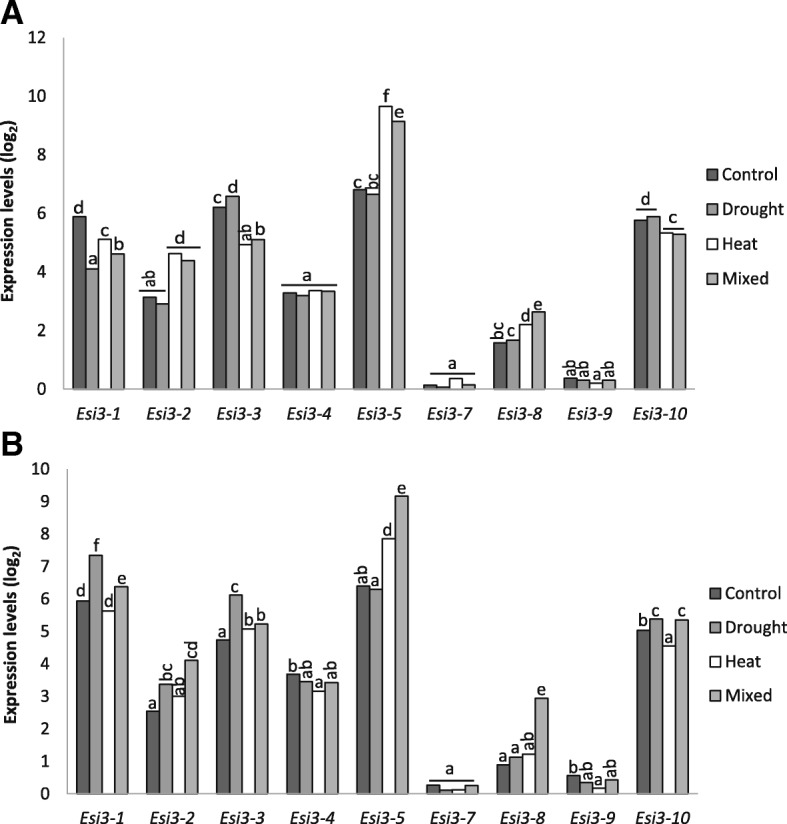
Long term drought stress. *Esi3* expression in response to drought, heat and both stresses combined compared between (**a**) Cappelli an efficient water use line and (**b**) Ofanto a water use inefficient line. Data is from Affymetrix microarray analysis. Values are in log_2_ units, thus an increase by 1 unit represents a two-fold increase, background of 3.4 subtracted from each value. Conditions were those described in Aprile et al., 2013 [31]; in brief, plants were stressed at booting stage by withholding water to 12.5% soil water content. Heat treatment was done by incremental increases to 40 °C, and the combined treatment used the same two conditions. Letters signify Duncan’s multiple range values

The *Esi3* gene family’s expression in leaf tissue in response to long term drought and high temperature stress was also analyzed in the microarray datasets for two durum wheat, *T. turgidum*, cultivars that differed in their degree of water use efficiency. The Cappelli cultivar has high water use efficiency and Ofanto has low water use efficiency [31]. The abiotic stress regimes were applied to soil-grown plants at booting stage. Though the changes of expression for the *Esi3* genes were generally similar in the two cultivars, there were a few striking differences (Fig. 3 and Additional file 11: Table S8). Cappelli had higher expression of *Esi3–3* under control conditions, though it had higher levels of induction in Ofanto in response to drought treatment. Nevertheless, the resulting levels of expression in Cappelli remained higher than Ofanto after being subjected to drought stress. *Esi3–1* was down-regulated by drought in Cappelli but up-regulated in Ofanto. The drought tolerant cultivar Cappelli had higher levels of expression of *Esi3–8* than the Ofanto cultivar under control, drought and heat treatments, but Ofanto showed *Esi3–8* expression levels similar to Cappelli under mixed drought and heat treatment. *Esi3–5* was more strongly induced by heat stress in Cappelli than in Ofanto, inversely *Esi3–10* was down-regulated by heat in Ofanto. *Esi3–8* was induced by combined drought and heat treatment in Ofanto. The response to mixed drought and heat treatments was complex, in the case of *Esi3–5* the induction due to heat was similar to the gene induction seen with heat and drought in Cappelli. In other cases, the induction due to treatments appears to be partially additive, as in Cappelli’s *Esi3–8*, or synergistic in the case of *Esi3–8* and *−5* in Ofanto (Fig. 3 and Additional file 11: Table S8). The differences in changes in *Esi3* expression in the two cultivars in response to heat and drought suggests that drought tolerant genotypes may have adaptive gene expression patterns.

**Fig. 4:**
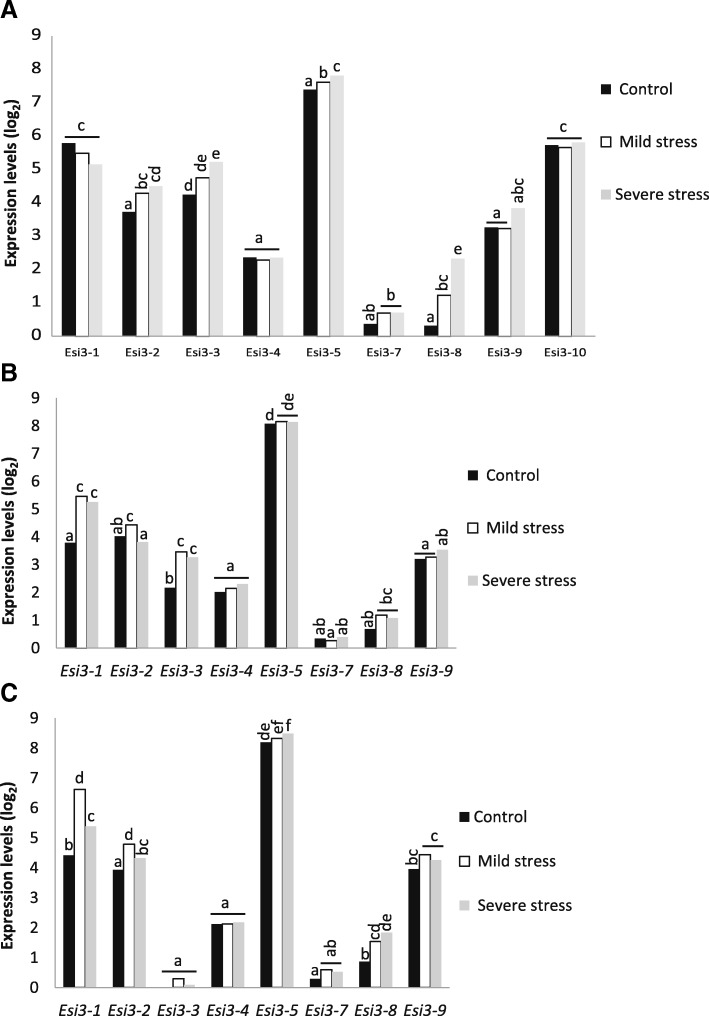
Long term drought stress. *Esi3* response to mild and severe drought stress in (**a**) the *T. turgidum* cultivar Creso, (**b**) the *T. aestivum* cutivar Chinese Spring (CS) and (**c**) a Chinese Spring line with a deletion in the 5AL chromosome (CS_5AL-10). *Esi3* expression was quantified with Affymetrix microarrays, *Esi3–10* is not represented on CS and CS_5AL-10 graphs since values are zero. Plants were grown as described by Aprile et al., 2009 [32]; briefly, soil grown plants were treated at anthesis at different levels of stress: Control field capacity 28% water content, mild stress 18% water content or severe stress at 12.5% water content. Background of 3.3 subtracted from all values, values are in log_2_ units. Letters signify Duncan’s multiple range values

*Esi3* gene expression patterns in response to drought were also compared between the *T. aestivum* cultivar Chinese Spring, the tetraploid *T. turgidum* cultivar Creso, and a Chinese Spring genetic line with the partial deletion of the long arm of chromosome 5AL (CS_5AL-10), based on microarray datasets of Aprile et al., 2009 [32]. The two species are closely related, the tetraploid *T. turgidum* being an ancestral species to *T. aestivum* and sharing the A and B genomes and genes with approximately 99% sequence identity. The *T. aestivum* cultivar Chinese Spring has higher water use efficiency than the *T. turgidum* cultivar Creso [32] and other studies have shown a general trend for *T. aestivum* to be more drought tolerant than *T. turgidum* [33]. The comparison demonstrated different responses to drought stress for *Esi3*s in these two closely related species (Fig. 4 and Additional file 12: Table S9). *Esi3–1* and *Esi3–3* had significantly higher constitutive levels of expression in Creso than in Chinese Spring. *Esi3–10* only has generally high expression levels in Creso and lacks expression in the other cultivars assayed. *Esi3–1* was up-regulated 1.4 fold in Chinese Spring in response to drought treatments, and moderately down-regulated in Creso (Fig. 4 and Additional file 12: Table S9). *Esi3–3* was more strongly up-regulated in Chinese Spring than in Creso. *Esi3–8* has moderately higher levels of mRNA in Chinese Spring in control conditions but it was more strongly induced in Creso than it was in Chinese Spring. Comparisons between Chinese Spring and a derivative genetic line with the 5AL chromosome partially deleted showed a similar induction of *Esi3–1* but showed expression for *Esi3–3* that was approximately 1/10th than that observed in other genotypes (Fig. 4 and Additional file 12: Table S9). The *Esi3–3* and *Esi3–10* genes are located on the long arm of chromosomes 5. Thus the deletion of chromosome 5AL may lower the level of expression for the homeologous group by nature of the deletion of the *Esi3–3-A* and *Esi3–10-A* gene copy. However, the high level of the reduction in expression suggests that it is due to the loss of a regulatory gene on chromosome 5AL that affects the expression of *Esi3–3* and *Esi3–10,* as this chromosome has been reported to contain several genes associated with the response to abiotic stress [34].

**Fig. 5:**
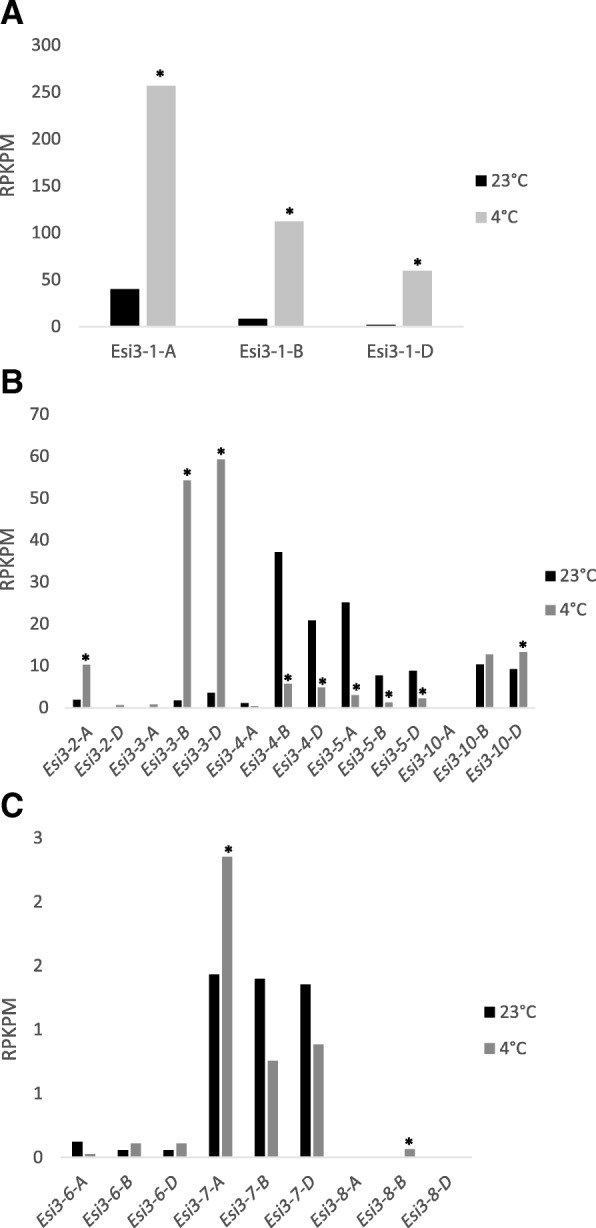
Short term cold treatment. Gene expression changes in *Esi3* in response to cold treatment at 4 °C. (**a**) *Esi3–1*, (**b**) *Esi3–2*, *−3, −4, -5*, and *−10* (**c**) *Esi3–6, −7,* and *−8* expression in response to cold treatment. The *Esi3s* are separated into three graphs due to large differences in gene expression corresponding to large differences in Y-axis values. *Esi3–9* is not graphed since there was no gene expression detected. * indicates significant changes in gene expression determined by a one-way ANOVA. Values are normalized to reads per kilobase per million (RPKPM)

Members of the *Esi3* gene family were also shown to have altered levels of expression in wheat leaf tissue in response to cold treatment (Fig. 5 and Additional file 13: Table S10). *Esi3–1-A, -B* and -*D* copies showed more than 6, 12 and 30-fold increase in expression levels, respectively, as well as high absolute levels of expression in leaf tissue of plants after they were shifted to growth from 23 °C to 4 °C for two weeks (Fig. 5 and Additional file 13: Table S10) [35]. *Esi3–2-A*, *Esi3–3-B, Esi3–3-D,* and *Esi3–10-D* also showed significant increased levels of expression though their absolute levels of expression were lower than *Esi3–1*’s. *Esi3–4-B* and -*D* as well as *Esi3–5-A, -B* and -*D* showed significant reduction in the levels of transcripts in response to cold treatment. In most cases homeologous copies of gene family members responded similarly, they tended to be either all induced or all repressed by cold treatment, though their absolute level of expression and the degree of change in expression within homeologous groups varied several fold; in multiple cases the changes in expression for some members of the homeologous groups were not statistically significant.

### *Fusarium graminearum* infection

**Fig. 6:**
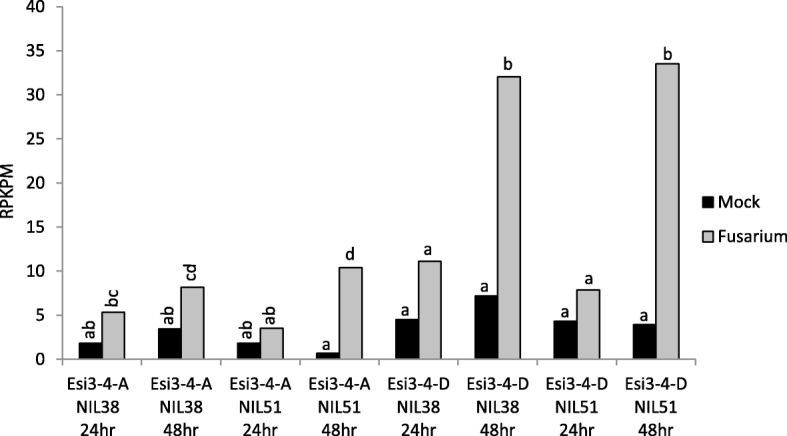
*Fusarium graminearum* inoculation. *Esi3–4-A* and *Esi3–4-D* changes in gene expression in NIL 38, a disease resistant line and NIL 51, a disease susceptible line, in response to *F. graminearum* inoculation after 24 and 48 h. Letters correspond to Duncan’s multiple range values to highlight significant differences in gene expression. Values are normalized to reads per kilobase per million (RPKPM)

Analysis of gene expression profiles by transcriptome sequencing for developing spikes inoculated with *Fusarium graminearum* [36] showed striking up-regulation for *Esi3–4-A* and *Esi3–4-D* at 24 and 48 h after inoculation. The up-regulation observed in both the *Fusarium* resistant line NIL 38 and the susceptible line NIL 51 was statistically significant, particularly at 48 h post inoculation (Fig. 6 and Additional file 14: Table S11). *Esi3–5-A, -B*, and -*D* as well as *Esi3–9-A* and -*B* showed higher absolute levels of expression than the *Esi3–4*s, but levels were not significantly changed in response to pathogen inoculation (Additional file 14: Table S11). However, the D copy for *Esi3–3* was up-regulated 6.5 fold 24 h post inoculation in the susceptible line NIL 51, however there was no induction in the resistant line NIL 38 (Additional file 14: Table S11). In fact, the only tissues in which *Esi3–9* expression was detected was in the spike and, as mentioned above, in the anthers.

## Discussion

The members of the *Esi3* gene family identified in *Triticum aestivum* in this study likely represent all members of the family. Whole genome sequencing for *T. aestivum* [22] and the extensive transcriptome databases for *T. aestivum* and other Triticum species were thoroughly searched for *Esi3*-like sequences. The resulting gene sequence set was verified in the recently released diploid genome for the D genome donor, *Ae. tauschii*, for which the full pseudomolecule sequences for the seven chromosomes are available [24]. The identification of homeologous genes in the A, B and D genomes for each family member provides additional confidence for the completeness of the searches. The set was compiled and refined through a series of iterative searches, and expanded when it was apparent that there may be members of the family missing from the dataset, such as an absent homeolog or a missing ortholog to a gene family member identified in closely relates species. Orthologs for all members of the *Esi3/RCI2/PMP3* gene family were also identified in closely related diploid species belonging to the Triticeae tribe, *Secale cereale*, *H. vulgare* and *Ae. tauschii*. The high degree of similarity among homeologous gene copies is similar to that described for other gene families in *Triticum aestivum*, alpha tubulins and caleosins, i.e. approximately 97% nucleotide sequence identity and 99–100% amino acid sequence identity for many homeologous sets [21, 37]. The degree of sequence similarity among homeologous gene copies is also similar to that among orthologs to the *Esi3* genes in other species of the Triticeae, *H. vulgare* and *Secale cereale*.

The comparison of the structure of the proteins predicted for the gene family members in *Triticum* indicate that species in the Triticeae contain a novel class of Esi3-like proteins in addition to the two groups of *Esi3/RCI2/PMP3* gene family members previously identified in other species [5]. *Esi3–9* constitutes a new class of proteins, Group III, characterized by long extensions of coding sequence on their N terminal ends. *Esi3–9*-like genes were also identified in the closely related species *H. vulgare, Secale cereale*, *Ae. tauschii* as well as *T. turgidum*. *Esi3*-like genes with the Group II structure were not identified in Arabidopsis nor in other monocot species surveyed which include *Z. mays*, *S. bicolor*, *O. sativa*, and *B. dystachion.* Other genes in the *Esi3* gene family can be classified into groups previously described in other species [5]. The *Esi3* genes were dispersed in the genome, however two pairs of genes were found to be localized in tandem. *Esi3–1* was localized near *Esi3–2,* and *Esi3–3* was localized near *Esi3–10* in the A, B and D genomes of *T. aestivum* as well as in *Ae. tauschii*. In *H. vulgare* the *Esi3–1* and *Esi3–2* genes are approximately 200 kb apart, but the species did not contain a copy of *Esi3–3* [23]. Other paralogous *Esi3* genes located on the same chromosomes are quite far from each other. The size of the *Esi3/RCI2/PMP3* gene family is similar in the other species studied; there were ten *Esi3/RCI2/PMP3* genes in *Ae. Tauschii* and *Secale cereale*, nine in *H. vulgare*, eight in *B. dictachyon,* and *A. thaliana*, seven in *Sorghum bicolor,* 11 in *Z. mays,* and 12 in *O. sativa*. The gene families for *A. thaliana, B. distachyon*, *O. sativa,* and *Z. mays* have been previously described [5].

The phylogenetic tree created with the nine species suggests that there have been independent radiations of the gene family among the monocot species (Additional file 15: Figure S2). Among the Triticeae most gene family members are common, with the exception of *Esi3–2* which is not present in the B genome of *T. aestivum* and *T. turgidum*, which suggests that it was deleted in these lineages (Additional file 16: Figure S3). Overall this pattern indicates that the ten-member gene family evolved to its current number before the divergence of the members of the Triticeae tribe. Though the dicot Arabidopsis has eight members of the *Esi3/RCI2/PMP3* gene family they do not have a close relationship with the gene family members in the Triticeae and other monocot species. The phylogenetic tree suggests that there have been a number of gene duplication events since the divergence of monocots and dicots and there have been additional gene duplication and gene losses since the divergence of *O. sativa, Z. mays, B. distachyon* and the Triticeae.

### Accuracy of gene annotation

The importance of manual annotation of gene families is apparent from the miss-annotation of the *Esi3/RCI2/PMP3* gene family members in several full genome annotation databases. This underscores the challenge of perfecting automated annotation of extensive DNA sequence sets. Automated gene annotations are particularly troublesome with genes with short coding sequences such as the *Esi3*s. Some miss-annotations detected in the whole genome database appear to be based on a preference for long open reading frames (ORFs). Several of the miss-annotated loci were identified with incorrect long ORFs that overlapped those of *Esi3/RCI2/PMP3* genes that would encode hypothetical proteins that have no similarity to those in other species. The genome for *S. cereal* is not annotated at Ensembl Plants [23]; these discrepancies are summarized in Additional file 3: Table S3. The annotations reported here relied heavily on the availability of transcript sequences from EST and TSA databases for gene identification and the demarcation of intron/exon junctions. Additional considerations included the detection of open reading frames, sequence and gene structural conservation among family members. We expected sequence similarity among homeologous groups of genes to be higher than 95% in most cases [21, 37]. When they are available, the transcript sequence is an important consideration in gene annotation. The transcripts in the TSA database were extremely useful because of the exceptional depth of the second generation sequences, as well as the accuracy of their assembly. However, they have a tendency to have excessively long 5′ and 3′ untranslated regions (UTR) sequences that do not appear to realistically represent the UTRs. These may be due to rare extended transcripts that are detected in the high depth of sequences available from second generation sequencing and that are included in the automated assemblies of transcripts. These extended UTR sequences are generally not seen in the EST databases which have traditionally contained sequences obtained from the Sanger sequencing methods on individual cDNA clones. It is advisable that these two types of transcript sequences remain separated in the public databases.

### Expression

Nearly all members of the *Esi3* gene family showed differential expression in response to abiotic or biotic stresses; this differential gene expression is taken as an indicator of the role these genes play in stress tolerance. The constitutive overexpression of select members of the *Esi3/RCI2/PMP3* gene family in Arabidopsis [10, 13–15] and *Nicotiana tobacum* [16] have demonstrated that these genes can increase abiotic stress tolerance and reduce Na^+^ accumulation in transgenic plants. Similarly, the expression of a large number of plant *Esi3/RCI2/PMP3* gene family members in Saccharomyces demonstrated that they can improve salt stress tolerance and complement the deletion of the yeast *PMP3* gene. The studies of Aprile et al., 2009 and 2013 [31, 32] compared wheat cultivars that differed in stress tolerance and water use efficiency and found expression patterns in which higher levels of expression or higher levels of induction paralleled the level of tolerance for the species or genotype. Long term drought treatment showed that *Esi3–1* was more strongly induced in the drought tolerant species *T. aestivum* than in the more sensitive *T. turgidum*. However, the opposite trend was seen for *Esi3–1* when comparing the high water use efficiency *T. turgidum* cultivar, Cappelli, with the low water use efficiency cultivar Ofanto. *Esi3–1* showed decreased expression in Cappelli in response to stress and increased expression in Ofanto [31].

The gene expression datasets explored to characterize *Esi3* gene family members in response to abiotic stresses included a wide range of stress treatments, such as short term and long term osmotic stress, heat, cold treatments and *Fusarium graminearum* inoculation. Plants that were used for these studies were grown under different conditions and analyzed at different stages of growth, including soil-grown plants at flowering stage, seedlings grown in sterile petri dishes and in the case of the Fusarium inoculation, during grain development. Thus the patterns of gene expression were quite diverse; nevertheless, nearly all members of the gene family were shown to be up-regulated in at least one stress condition.

Experiments that were assayed by transcriptome sequencing facilitated the comparison of expression of individual homeologs. In most cases the members of the same homeologous set showed similar gene expression changes, though in some cases these changes were not statistically significant. We point out that most gene expression assays for stress treatments were measured on leaf tissue, whereas studies of tissue-specific expression showed that the *Esi3* gene family members were differentially expressed over a broad range of tissues and developmental stages. If these tissues were evaluated separately a more complex pattern of gene expression in response to stress may be recognized. Indeed, the initial description of *Esi3* differential gene expression was described in the relatively strong induction by salt treatment in the roots of the tolerant species *Lophopyrum elongatum* for the *Esi3–1* gene family member [1].

The *Esi3–9*s had the most unique structural features among the gene family members; they also have the most unique expression pattern. Transcripts for these genes were detected in very few datasets, namely in an RNA-seq datasets from anthers in Triticale and the anthers from the Azhurnaya Spring Wheat [21, 28] and in developing seed heads in the experiments that characterized the response to *Fusarium* inoculation of developing spikes.

The widespread differential gene expression of the *Esi3* gene family members in Triticum species in response to abiotic stress suggests that nearly all members of the family may contribute to abiotic stress tolerance. This would merit further investigation through transgenic studies or by comparative expression analysis in genotypes with differing degrees of stress tolerance.

## Conclusions

There are twenty-nine *Esi3/RCI2/PMP3* gene family members with ten paralogous groups, each with three homeologous copies in *T. aestivum* except for *Esi3–2* for which no B copy was identified. *Esi3–9*s have an extended N terminal therefore placing it in a group on its own, which has been designated Group III. The other *Esi3*s fall within Group I or II based on amino acid length and properties [5]. The gene family members were manually curated and compared to sequences that were annotated automatically and the manually curated sequences were more accurate. This study highlights the importance of manually curating sequences to elucidate gene families and their interrelationships, and for the improvement of automated annotation methods. The *T. aestivum Esi3*s share homology with *Esi3/RCI2/PMP3* genes from *A. thaliana*, *Z. mays*, *O. sativa*, *B. distachyon*, *Sorghum bicolor, Ae. tauschii*, *H. vulgare*, and *Secale cereale*. The ten paralogous *Esi3*s have differential expression in response to a variety of abiotic stresses and in response to infection with *F. graminearum*. The homeologous copies also displayed varied responses to abiotic and biotic stress, suggesting that the *Esi3* family members play a role in stress tolerance.

## Methods

### *Esi3* gene sequence retrieval

The *Esi3* cDNA sequence from *L. elongatum* (GB Accession U00966.1) and the *Lti6A* and *Lti6B* genes from *Oryza sativa* [8] were used to search the National Center for Biological Information (NCBI) Transcriptome Shotgun Assembly (TSA) databases for *T. aestivum* and *T. monococcum* by Blastn and tBlastn; duplicate hits were eliminated. A total of ten paralogous gene sequences were found, and these were used to search the NCBI EST database and the International Wheat Genome Sequencing Consortium (IWGSC) [22] wheat survey sequences (WSS) versions 2 and 3 of individual chromosome arms. The sequences were also re-confirmed using the IWGSC whole genome assembly RefSeq v1.0 [38, 39]. The latter were used to make chromosomal arm assignments to individual homeologous copies of the gene family members from the A, B and D genomes of *T. aestivum*. Novel sequences identified in either of these databases were iteratively used to query the TSA and EST databases to verify the sequence and delimit exon/intron junctions in the gene sequence. In cases where there was discrepancy between sequences from different databases, contigs were re-assembled with *T. aestivum* EST sequences that shared a minimum of 99% identity using the CAP3 assembly program at prabi [40]. The paralogous set of *Esi3* gene family members were used to identify homologs in other monocotyledonous species, including *Secale cereale, H. vulgare, and Sorghum bicolor* in the GenBank nucleotide collection, TSA and EST databases. *Ae. tauschii* sequences were obtained from the NCBI TSA and nucleotide collection databases and from the *Ae. tauschii* Genome release of unannotated pseudomolecule sequences [25]. *Z. mays* sequences for *Esi3/RCI2/PMP3* genes were taken from those described by Fu et al., 2012 and Zhao et al., 2014 [10, 18]; *O. sativa* genes were taken from those reported by Morsy et al., 2005 [8] and Medina et al., 2007 [4]. Sequences of *Esi3*-like genes for *B. distachyon* were those reported by Rocha [5]; those for Arabidopsis were taken from Capel et al., 1997 [6], and Medina et al., 2007 [4].

### Phylogenetic analysis

Phylogenetic trees were constructed using Molecular Evolutionary Genetics Analysis (MEGA) version 7 [41]. The *Esi3* homeolog phylogenetic tree was constructed using the nucleotide sequences of the coding regions from all gene family members from *T. aestivum*. The Maximum Likelihood method was used based on the Jukes-Cantor model [42] with the same parameters used by Khalil et al., 2014 [21]. The analysis involved 29 nucleotide sequences. Codon positions included were 1st + 2nd + 3rd + Noncoding. All positions with less than 95% site coverage were eliminated. That is, fewer than 5% alignment gaps, missing data, and ambiguous bases were allowed at any position. The percentage of trees in which the associated taxa clustered together is shown next to the branches. There were a total of 165 positions in the final dataset.

The phylogenetic tree and sequence alignment of the *Esi3* homologs in nine species was done using the amino acid sequences. The sequences were aligned using MUSCLE [43] and the phylogenetic tree was constructed using ten paralogous *Esi3* genes from *T. aestivum*, one representative from each homeologous group and the full set of protein sequences from the diploid species *Ae. tauschii, H. vulgare*, *B. distachyon*, *Secale cereale*, *O. sativa, Z. mays, Sorghum bicolor* and *A. thaliana*. The evolutionary history was inferred using the Maximum Likelihood method based on the Whelan And Goldman model (WAG) [44]. A discrete Gamma distribution was used to model evolutionary rate differences among sites (5 categories (+G, parameter =1.5141)). The analysis involved 85 amino acid sequences. All positions containing gaps and missing data were eliminated. There were a total of 54 positions in the final dataset.

### *Esi3* expression analysis

The *Esi3* gene expression levels in response to abiotic and biotic stresses including drought, heat, combined heat and drought, low temperature and *Fusarium graminearum* infection were determined using Illumina RNA-sequencing libraries listed in the NCBI Sequence Read Archive (SRA) database and then retrieved from the European Nucleotide Archive at EMBL-EBI [45] Datasets used for gene expression analysis are listed in Additional file 17: Table S12. The datasets in FASTQ format were converted to FASTA using FASTX Toolkit 0.0.13.2 [46]. Due to the high sequence similarity found within the coding region of the *T. aestivum Esi3* sequences, the 3’ UTRs from the EST sequences were used to search the datasets using CD-HIT-EST-2D biological sequence clustering algorithm [47]. The CD-HIT-EST-2D parameters were set to default with the exception of a word size of 5 (*n* = 5), similarity cut-off of 99% (−c 0.99) and a memory limit of 32G (−M 32000 Mbytes). The expression was normalized to reads per kilobase per million (RPKPM) to normalize for differing library sizes and different 3’ UTR lengths among the gene transcripts. The RPKPM was calculated as the number of reads in a library with a minimum of 99% identity to the query sequence/the length of the query sequence/ number of reads in a library/10^6^.

Affymetrix microarray 61 k RMA normalized datasets (log_2_ units) [29] for different wheat tissues at different times of development and to assay gene expression in response to drought, heat, and combined drought and heat stress were retrieved from the PLEXdb database [48]. The differences in fold expression were deduced by comparing the levels of treated samples relative to the levels found within the control measured by fluorescence intensity. The levels of *Esi3* gene expression in response to drought was analyzed using the dataset from Aprile et al., 2009 [32]. The effects of drought, heat and combined stress was analyzed using the dataset from Aprile et al., 2013 [31]. The *Esi3* expression levels in different tissue types were analyzed using a dataset from Schreiber et al., 2009 [29]. Homeologous gene copies in *T. aestivum* cannot be distinguished by the microarray analysis; results were analyzed for the wheat *Esi3* paralogous genes, which represent the combined set of three homeologous genes.

Expression data analysis of the *Esi3* gene family across seventy-one tissue types reported in [28] was retrieved from the wheat eFP browser at the University of Toronto BAR [49]. The identifiers for all *Esi3* gene family members were retrieved from the Ensembl Plants and listed in Additional file 1: Table S1. Data was not available for *Esi3–1-D* as well as for *Esi3–10-A* since the latter has not been identified on Ensembl Plants [23].

### Statistical analysis

The significance of differences in *Esi3* gene expression, in the RNA-seq datasets, was analyzed using a two-way ANOVA to test for significant differences in expression between genotypes in response to treatments and for genotype x treatment interaction effects. Subsequently, the data was analyzed using a one-way ANOVA test with a Duncan’s multiple range post hoc test to determine the differences in *Esi3* induction between the A, B, and D copies in response to stress or in differing tissue types. One-way ANOVA was used to test the significance of the differences in *Esi3* gene expression from microarray data. Duncan’s multiple range post hoc test was used to determine the significance of differences in gene expression in response to different stress conditions.

## Additional files

Additional file 1: **Table S1.** Esi3/RCI2/PMP3 identifiers from Ensembl Plants and amino acid sequence comparison with current annotations of *Triticum aestivum*. (XLSX 11 kb)

Additional file 2: **Table S2.** Sequence identity matrix for nucleic acid and amino acid sequences among members of the* Esi3/RCI2/PMP3* gene family in *Triticum aestivum*. (XLSX 18 kb)

Additional file 3: **Table S3.** *Esi3/RCI2/PMP3* gene family members in eight species with NCBI accession numbers and comparisons to Ensembl Plants annotations. (XLSX 15 kb)

Additional file 4: Esi3/RCI2/PMP3 protein sequences for the eight species. (DOCX 17 kb)

Additional file 5: *Triticum aestivum* *Esi3* protein and nucleotide coding regions. (DOCX 18 kb)

Additional file 6 **Table S4.** *Esi3/RCI2/PMP3* gene expression levels in five tissue types of *T. aestivum.* (XLSX 18 kb)

Additional file 7: **Table S5.** *Esi3/RCI2/PMP3* expression in *T. aestivum* in seventy-one tissue types. (XLSX 63 kb)

Additional file 8: **Figure S1.** Tissue specific expression of the *Esi3* genes in thirteen *T. aestivum* tissue types assayed by microarray. (PDF 298 kb)

Additional file 9: **Table S6.** *Esi3/RCI2/PMP3* tissue-specific expression assayed by microarray analysis. (XLSX 13 kb)

Additional file 10: **Table S7.** *Esi3/RCI2/PMP*3 gene expression in T. aestivum in response to drought and heat stress. (XLSX 15 kb)

Additional file 11: **Table S8.** *Esi3/RCI2/PMP3* expression in response to drought in Cappelli, a water use efficient line, and Ofanto, a water use inefficient line of T. turgidum. (XLSX 12 kb)

Additional file 12: **Table S9.** *Esi3/RCI2/PMP3* response to drought in the hexaploid Chinese Spring, the tetraploid Creso, and a Chinese Spring chromosome deletion line. (XLSX 12 kb)

Additional file 13: **Table S10.** *Esi3/RCI2/PMP3* gene expression in cold treated wheat seedlings. (XLSX 12 kb)

Additional file 14: **Table S11.** *Esi3/RCI2/PMP3* gene expression in wheat spikes in response to *Fusarium graminearum* inoculation. (XLSX 22 kb)

Additional file 15: **Figure S2.** Molecular Phylogenetic analysis of the Esi3/RCI2/PMP3 protein sequences from nine species. (PDF 384 kb)

Additional file 16: **Figure S3.** Molecular phylogenetic analysis of the *Esi3* genes from *T. aestivum*. (PDF 244 kb)

Additional file 17: **Table S12.** *Triticum aestivum* RNA-seq datasets used to compare *Esi3/RCI2/PMP3* gene expression levels. (XLSX 15 kb)
