# Supplementary material for: Characterization of the Esi3/RCI2/PMP3 gene family in the Triticeae
Source: BMC Genomics. 2018 Dec 11;19:898. doi: 10.1186/s12864-018-5311-8 (PMC6288971; doi:10.1186/s12864-018-5311-8)
Supplement: Supplementary file 4 — Esi3/RCI2/PMP3 protein sequences for the eight species. (DOCX 17 kb) [file 12864_2018_5311_MOESM4_ESM.docx]

***Brachypodium distachyon***

>*BdRCI2-1*_XP_003563330.1 MSSSGGCSTCLETIFAAVLPPLGVFFRYGCCSSEFFISLLLTALGYVPGIAYSVWVILKTAPEPPGIDGDRPYYILA

>*BdRCI2-2*_XP_003568974.1

MAGTANCIDIILAIILPPLGVFLKFGCGHEFWICLLLTFLGYIPGIIYAIYAITK

>*BdRCI2-3*_XP_003557909.1

MADNTATFIDLILAIILPPLGVFLKYGCEIEFWICLVLSFFGYLPGIIYAVWVIVK

>*BdRCI2-4*­_XP_003558174.1

MASATFLEVILAIILPPVGVFLRYGLGVEFWICLLLTILGYIPGIIYAVYVLVA

>*BdRCI2-5*_XP_003578630.1

MASGGCCTFLEILLAIFLPPLGVFLHYGCCSMEFCICLLLTILGYIPGIIYAIYVLVALDSEERHREYYTLA

>*BdRCI2-6*_XP_003564192.1

MGLGSCCCRCLEILCAILLPPLGVCLRHGCCSMEFWISVLLTILGYLPGVLYAAYVILSVDPDRVRRRGHDDDDDYIYVA

>*BdRCI2-7*_XP_014752685.1

MADEGTANCIDIILAIILPPLGVFFKFACGIEFWICLLLTFFGYLPGIIYAVWVITR

>*BdRCI2-8*_XP_014754368.1

MSNSTEKCVSIVLAIILPPLGVLLKFGCQTEFWLCLLLTLFGYLPGIIYAVYVLTK

***Hordeum vulgare***

>HvEsi3-1_BAJ85904.1

MGSATVLEVILAIILPPVGVFLRYKLGVEFWICLLLTILGYIPGIIYAVYVLVV

>HvBlt101-2_BAJ89049.1

MASATFIEVILAIILPPVGVFLRYGLAVEFWICLLLTLLGYIPGIIYAVYVLVA

>HvEsi3-4_BAK03378.1

MAGTANCIDIILAIILPPLGVFLKFGCGHEFWICLLLTFLGYIPGIIYAIYAITK

>HvEsi3-5_BAJ88867.1

MADEGTANCIDIILAIILPPLGVFFKFACGIEFWICLLLTFFGYLPGIIYAVWVITK

>HvEsi3-6_BAK03743.1

MSSGGCSTCLEIIFAAVLPPLGVFFRYGWCSSEFFISLPLTMLGYVPGIIYSVYVILKTPPELPSIDGDRPYYILA

>HvEsi3-7_DK716258.1

MGMCSCCCRCLEIMCAILLPPLGVCLRHGCCSMEFWISVLLTILGYLPGVLYAAYVICSVDPERVRRRGDSDDDYIYVA

>HvEsi3-8_BG416092.2

MGSETFVEILLAILLPPVGVFLRYGIGMEFWICLLLTLLGYIPGIIYAIFVLVA

>HvEsi3-9_BAK07288.1

MAETAAIAPPPQPMAESATAAPPQPMAGNATTAAVVVVVPPPSPPDNTMTFLCLLIAIFLPPLGVFIKYNCEVEFWI

CLVLTFFGYFPGVIYAIWVIVKP

>HvEsi3-10_ BAJ90014.1

MASRSCTFLEILFAIILPPLGVFLRFGCCSMEFCICLLLTILGYIPGIIYAVYVLVALGSEDRDRDYDTLA

***Secale cereale***

>ScEsi3-1_GCJW01020680.1

MGSATVLEVILAIILPPVGVFLRYKLGVEFWICLLLTILGYIPGIIYAVYVLVV

>ScEsi3-2_GCJW01023827.1

MASATFIEVILAIILPPVGVFLRYGLAVEFWICLLLTLLGYIPGIIYAVYVLVA

>ScEsi3-3_GCJW01020158.1

MASQGCTFLEILIAVLLPPLGVFLRYGCCSMEFLICLLLTILGYIPGIIYAVYVLVSHGSASQERDYDALA

>ScEsi3-4_GCJW01019226.1

MAGTANCIDIILAIILPPLGVFLKFGCGHEFWICLLLTFLGYIPGIIYAIYAITK

>ScEsi3-5_GCJW01023929.1

MADEGTANCIDIILAIILPPLGVFFKFACGIEFWICLLLTFFGYLPGIIYAVWVITK

>ScEsi3-6_GCJW01020650.1

MSYSGGCSTCLEIVFAAVLPPLGVFFRYGWCSSEFFISLPLTMLGYPGIIYSVYVILKTPPELPSIDGERPYYILA

>ScEsi3-7_GCJW01023828.1

MGMCSCCCRCLEILCAILLPPLGVCLRHGCCSMEFWISVLLTILGYLPGVLYAAYVICSVDPDRVRRRDDDYIYVA

>ScEsi3-8_GCJW01021120.1

MGSETFVEILLAILLPPVGVFLRYGVGVFWICLLLTVLGYIPXIIYAIFVLVA

>ScEsi3-9_GCJW01018134.1

MAESAVIAPPPQPTAPPQPMAESATAPPSQPMAPPQPMAENATAAPPQPMAENATATVVVVVPPPPPDSTTTFLCLILAFFIPPLGVFIKYECEVEFWICLVLTFLAYAPGIIYAVWVIVKK

>ScEsi3-10_ GCJW01020353.1

MASRSCTFLEILLAIFLPPLGVFLHYGCCSMEFCICLLLTILGYIPGIIYAVYVLVALGSEDRDRDYDNLA

***Oryza sativa***

>OsRCI2-1_EEE54356.1

MSDGTANCIDILIAIILPPLGVFLKFGCKVEFWLCLLLTFFGYLPGIIYAVYAITK

>OsRCI2-2_ AP014957.1

MTNHHHHHHHHDDDHKVGANFCNILLSIILPPFAIGVVVGCKKEFWICLLLTCLGYLPGIIYAICIISGAHIHD

>OsRCI2-3_AAG46140.1

MADRPPAMADRTATFVDLVIAIILPPLGVFLKVGCEIEFWICLLLTFLGYFPGIIYAVWVIVNH

>OsRCI2-4_EAY90184.1

MADRTATFVDLVIAIILPPLGVFLKVGCEIKFWICLLLSFFGYLPGIIYAVWVIVNH

>OsRCI2-5_XP_015633211.1

MASATFLEVLLAIFLPPVGVFLRYGLGIEFWIDLLLTILGYIPGIIYAVYVLVA

>OsLTI6A_XP_015647973.1

MADSTATCIDIILAIILPPLGVFFKFGCGIEFWICLLLTFFGYLPGIIYAVWVITK

>OsLTI6B_XP_015640253.1

MAGTANCIDILIAIILPPLGVFLKFGCGHEFWICLLLTFLGYIPGIIYAIYAITK

>OsRCI2-7_XP_015637682.1

MGSETFLEILLAILLPPLGVFLRYGIGMEFWIALLLTILGYLPGIIYAVYVLVA

>OsRCI2-8_XP_015643303.1

MGCCCRCLEILCAILLPPLGVCLRHGCCTMEFWISVLLTILGYLPGVLYAVYVIVSVDPDRERRRRVDPDEYIYVA

>OsRCI2-9_EAZ01907.1

MSYSGGCSTCLETIFSVVLPPLGVFFRYGFCSSEFVVSSALTALFYVPGIVYSVWVVILKTPPEPPGIDGERPYYILA

>OsRCI2-11_XP_015612003.1

MASGRCCTFLEILLAIILPPLGVFLRFGCCSMEFCICLLLTILGYVPGIIYAVYVLVALDSDQYQREYHTLA

>OsRCI2-12_BAF24778.1

MGHFMMDDQNIFAWLCTSCVLCCFMGCAYIFYIIVTIILPPLPVFIRHHCEVSQICRFYLVSDVLKRNKLVFISCYSFRCLA

***Zea mays***

>ZmPMP3-1­_EU364508.1

MSEGTANCVDILIAIILPPLGVFLKYGCGHEFWICLLLTFLGYIPGIIYAIYAITKNN

>ZmPMP3-2­_EU959002.1

MADGRCCTFLEILFAIILPPLGVFLRFGCCRIEFCICLLLTILGYVPGIIYAIYVLVALDSDQHEREYYTLA

>ZmPMP3-3_EU962407.1

MGLCSCCCRCLELLCSVLLPPLGVCLRHGCCSMEFWISVLLTILGYLPGVLYAVYVICSVDPHRHRDDDDYVYVA

>ZmPMP3-4_EU954642.1

MASATFLEVLLAIFLPPVGVFLRYGCGVEFWIDLLLTVLGYIPGIIYAVYVLVA

>ZmPMP3-5_EU975274.1

MSDGTATCIDIILAIILPPLGVFFKFGCGVEFWICLILTFFGYLPGIIYAVWAITK

>ZmPMP3-6_EU976341.1

MGSETFVEILLAILLPPVGVFLRYGIGVEFWICLLLTILGYIPGIIYAVYVLVA

>ZmPMP3-7_EU955642.1

MKEGTANCIDILIAIILPPLGVFLKFGCKVEFWLCLLLTFLAYLPGIIYAIYVITKD

>ZmPMP3-8_EU971491.1

MKEGTANCVDILIAIILPPLGVFLKFGCKVEFWLCLLLTFLAYLPGIIYAIYAITKD

>ZmRCI2-3_XP_020404221.1

MSDGTATCVDIILAIILPPLGVFFKFGCGAEFWICLILTFFGYLPGIIYAVWVITMFFF

>ZmRCI2-8_NP_001151922.1

MSSGGCSTCLEVIFAAILPPLGVFFRYGCCSSEFFVSLLLTLLCYVPGVAYSLYVILRTPPEPPGIDGERPYDMLA

>ZmRCI2-9_XP_020399959.1

MADNMVTLVRLVLAIILPPLGVFLKHGLKIEFWICLLLCFFGYLPGVIYAVWVIIRKEDD

***Arabidopsis thaliana***

>AtRCI2A­_NP_187239.1

MSTATFVDIIIAILLPPLGVFLRFGCGVEFWICLVLTLLGYIPGIIYAIYVLTK

>AtRCI2B_NP_187240.1

MSTATFVEIILAIILPPLGVFLKFGCKVEFWICLILTLFGYLPGILYALYIITK

>AtRCI2C_NP_176067.1

MGSFLEVLCAIFIPPVGVFLRYGLGLEFWVCLLLTLFAFIPGLIYAIYVLTK

>AtRCI2D_NP_179982.1

MASSCELCCEIFIAILLPPVGVCLRHGCCTVEFFICLILTCLGYLPGIIYAIYAICFLHRDEYFDEYRRPIYYVA

>AtRCI2E_NP_194794.1

MASNMEVFCEILIAILLPPLGVCLKRGCCTVEFLICLVLTILGYIPGIIYALYVIVFQNREGSTELGAPLNSA

>AtRCI2F_NP_194795.1

MPSNCEILCEIIIAILLPPLGVCFRKGCCTVEFLICLVLTILGYVPGIIYAIYVIVFQHREEYFDEYRRPIYSA

>AtRCI2G_NP_974629.1

MANGCEICCEIMIAILIPPLGVCLRHGCCTTEFMICLILTLLGYVPGIIYALYAIVYVDRDQFFDEYRRPLFYAQSP

>AtRCI2H_NP_565897.1

MGSETFLEIILAILLPPVGVFLRYGCGVEFWICLLLTILGYIPGIIYAIYVLVG

***Sorghum bicolor***

>SbEsi3-1_XP_002440508.1

MGSETFLEILLAILLPPVGVFLRYGIGVEFWICLLLTILGYIPGIIYAVYVLVA

>SbEsi3-2_XP_002465426.1

MASATFLEVLLAIFLPPVGVFLRYGCGVEFWIDLLLTVLGYIPGIIYALYVLVA

>SbEsi3-3_ XP_002460652.1

MADGRCCTFLEILLAIILPPLGVFLRFGCCRIEFCICLLLTILGYIPGMIYAIYVLVALDSDRHEREYYTLA

>SbEsi3-4_XP_021309366.1

MSNSDGTATCIDIILAIILPPLGVFFKFGCGVEFWICLILTFFGYLPGIIYAVWAITK

>SbEsi3-5_XP_002437373.1

MSSGGCSTCLEVIFAVVLPPLGVFFRYGCCSSEFFISLLLTVLCYVPGIVYSLYVILRTPPEPPGIDGERPYDMLA

>SbEsi3-6_XP_002437973.1

MGLCSCCCRCLELLCSVLLPPLGVCLRHGCCSLEFWISVLLTILGYLPGVLYAVYVICSVDPHRHRDPDDDYVYVA

>SbEsi3-7_XP_002465219.1

MADDNTVTLVRLILAIILPPLGVFLKHGLKLEFWICLILSFFAYLPGIIYAVWVIIKKDDD

***Aegilops tauschii***

>AetEsi3-1_XP_020150593.1

MGSATVLEVILAIILPPVGVFLRYKLGVEFWICLLLTILGYIPGIIYAVYVLVV

>AetEsi3-2­_XP_020150605.1

MASATFIEVILAIILPPVGVFLRYGLAVEFWICLLLTLLGYIPGIIYAVYVLVA

>AetEsi3-3­_ XP_020168538.1

MASRSCTFLEILLAVILPPLGVFLRYGCCSMEFLICLLLTILGYIPGIIYAVYVLVAHGSASEESGRDYDALA

>AetEsi3-4­_XP_020190487.1

MAGTANCIDIILAIILPPLGVFLKFGCGHEFWICLLLTFLGYIPGIIYAIYAITK

>AetEsi3-5­_XM_020319125.1

MADEGTANCIDIILAIILPPLGVFFKFACGIEFWICLLLTFFGYLPGIIYAVWVITK

>AetEsi3-6_XP_020198926.1

MSYSGGCSTCLEIVFAAVLPPLGVFFRYGWCSSEFFISLPLTILGYVPGIIYSVYVILKTPPELPSIDGDRPYYILA

>AetEsi3-7­_XP_020151310.1

MGLCSCCCRCLEILCAILLPPLGVCLRHGCCSMEFWISVLLTILGYLPGVLYAAYVICSVDPDRVRRRDDDYIYVA

>AetEsi3-8_XP_020178067.1

MGSETFVEILLAILLPPVGVFLRYGIGVEFWICLLLTLLGYIPGIIYAIFVLVA

>AetEsi3-9_XP_020160007.1

MAESAAIAPPPQPMAPPQPVEENATAAPPQPMAPPQPMAENATAAPPQPMAENATVVVVVPPPPPDGTTTFLCLILAFFIPPLGVFLKYKCEIEFWICLILTFLAYAPGIIYAVWVIVK

>AetEsi3-10_XP_020168537.1

MASRSCTFLEILLAIILPPLGVFLHYGCCSMEFCICLLLTILGYIPGIIYAVYVLVALGSEERDRDYDTLA
