# Supplementary material for: Characterization of the Esi3/RCI2/PMP3 gene family in the Triticeae
Source: BMC Genomics. 2018 Dec 11;19:898. doi: 10.1186/s12864-018-5311-8 (PMC6288971; doi:10.1186/s12864-018-5311-8)
Supplement: Supplementary file 5 — Triticum aestivum Esi3 protein and nucleotide coding regions. (DOCX 18 kb) [file 12864_2018_5311_MOESM5_ESM.docx]

**Amino Acid Sequences**

>TaEsi3-1-A_JP881209.1

MGSATVLEVILAIILPPVGVFLRYKLGVEFWICLLLTILGYIPGIIYAVYVLVV

>TaEsi3-1-B_JZ888897.1

MGSATVLEVILAIILPPVGVFLRYKLGVEFWICLLLTILGYIPGIIYAVYVLVV

>TaEsi3-1-D_JP881207.1

MGSATVLEVILAIILPPVGVFLRYKLGVEFWICLLLTILGYIPGIIYAVYVLVV

>TaEsi3-2-A_HAAB01071723.1

MASATFIEVILAIILPPVGVFLRYGLAVEFWICLLLTLLGYIPGIIYAVYVLVA

>TaEsi3-2-D_HAAB01071724.1

MASATFIEVILAIILPPVGVFLRYGLAVEFWICLLLTLLGYIPGIIYAVYVLVA

>TaEsi3-3-A_CV767975.1

MASRSCTFVEILLAVILPPLGVFLRYGCCSMEFLICLLLTILGYIPGIIYAIYVLVAHGSASEESGKDYDALA

>TaEsi3-3-B_CJ925039.1

MASRSCTFVEILLAVILPPLGVFLRYGCCSMEFLICLLLTILGYIPGIIYAVYVLVAHGSASEESGRDYDALG

>TaEsi3-3-D_CJ595273.1 CJ591008.1 CJ615800.1

MASRSCTFLEILLAVILPPLGVFLRYGCCSMEFLICLLLTILGYIPGIIYAVYVLVAHGSASEESGRDYDALA-

>TaEsi3-4-A_CA665474.1

MAGTANCIDIILAIILPPLGVFLKFGCGHEFWICLLLTFLGYIPGIIYAIYAITK

>TaEsi3-4-B_CJ901293.1

MAGTANCIDIILAIILPPLGVFLKFGCGHEFWICLLLTFLGYIPGIIYAIYAITK

>TaEsi3-4-D_JV989019.1

MAGTANCIDIILAIILPPLGVFLKFGCGHEFWICLLLTFLGYIPGIIYAIYAITK

>TaEsi3-5-A_HAAB01033003.1

MADEGTANCIDIILAIILPPLGVFFKFACGIEFWICLLLTFFGYLPGIIYAVWVITK

>TaEsi3-5-B_JV846264.1

MADEGTANCIDIILAIILPPLGVFFKFACGIEFWICLLLTFFGYLPGIIYAVWVITK

>TaEsi3-5-D_HP629889.1

MADEGTANCIDIILAIILPPLGVFFKFACGIEFWICLLLTFFGYLPGIIYAVWVITK

>TaEsi3-6-A_CJ671046.1

MSSGGCSTCLEIIFAAVLPPLGVFFRYGWCSSEFFISLPLTILGYVPGIIYSVYVILKTPPELPSIDGERPYYILA

>TaEsi3-6-B_CJ562290.1

MSYSGGCSTCLEIVFAAVLPPLGVFFRYGWCSSEFFISLPLTMLGYVPGIIYSVYVILKTPPELPSIDGERPYYILA

>TaEsi3-6-D_CJ559253.1

MSYSGGCSTCLEIVFAAVLPPLGVFFRYGWCSSEFFISLPLTILGYVPGIIYSVYVILKTPPELPSIDGDRPYYILA

>TaEsi3-7-A_BJ261574.1

MGLCSCCCRCLEIMCAILLPPLGVCLRHGCCSMEFWISVLLTILGYLPGVLYAAYVICSVDPDRVRRHDDDYIYVA

>TaEsi3-7-B_CJ725702.1

MGLCSCCCRCLEIMCAILLPPLGVCLRHGCCSMEFWISVLLTILGYLPGVLYAAYVICSVDPDRVRRRGDSDDDYIYVA

>TaEsi3-7-D_CJ825516.1

MGLCSCCCRCLEILCAILLPPLGVCLRHGCCSMEFWISVLLTILGYLPGVLYAAYVICSVDPDRVRRRDDDYIYVA

>TaEsi3-8-A_CD909025.1

MGSETFVEILLAILLPPVGVFLRYGIGVEFWICLLLTVLGYIPGIIYAIFVLVA

>TaEsi3-8-B_HAAB01084472.1

MGSETFVEILLAILLPPVGVFLRYGIGVEFWICLLLTVLGYIPGIIYAIFVLVA

>TaEsi3-8-D_ CJ648786.1

MGSETFVEILLAILLPPVGVFLRYGIGVEFWICLLLTVLGYIPGIIYAIFVLVA

>TaEsi3-9-A_HAAB01083453.1

MAETAAIAPPPQPMAPPQPVEENATAAPSQPMAPLQPMAESATVVVVVPPPPPDGTTTFLCLILAFFIPPLGVFLKYKCEIEFWICLILTFLAYAPGIIYAVWVIVK

>TaEsi3-9-B_BJ243843

MAESAVIAPPLQPMTPPQPMAENATEAPPQPMAPPQQMAENATAAPPPQPMAENATAAPPPQPMVENATVVVVVPPPPPDSTTTFLCLILAFFLPPLGVFLKYKCEIEFWICLILTFLAYAPGIIYAVWVIVK

>TaEsi3-9-D_BJ243706.1

MAESTAIAPPPQPMAPPQPVEENATAAPPQPMAPPQPMAENATAAPPQPMAENATVVVVVPPPPPDGTTTFLCLILAFFIPPLGVFLKYKCEIEFWICLILTFLAYAPGIIYAVWVIVK

>TaEsi3-10-A_HX161660.1 HX161634.1 CJ823027.1 CA645450.1

MASRSCTFLEILLAIILPPLGVFLHYGCCSMEFCICLLLTILGYIPGIIYAVYMLVALGSEERDRDYNTLA-

>TaEsi3-10-B_CA611646.1

MASRSCTFLEILLAIILPPLGVFLHYGCCSMEFCICLLLTILGYIPGIIYAVYVLVALGSEERDRDYDTLA-

>TaEsi3-10-D_BJ278420.1

MASRSCTFLEILLAIILPPLGVFLHYGCCSMEFCICLLLTILGYIPGIIYAVYVLVALGSEERDRDYDTLA-

**Coding Sequences**

>TaEsi3-1-A_JP881209.1

ATGGGCTCGGCAACAGTCCTGGAGGTGATCCTCGCCATCATCCTGCCTCCCGTCGGCGTCTTCCTGCGCTACAAACTCGGCGTGGAGTTCTGGATCTGTCTCTTGCTGACCATACTGGGGTACATACCGGGGATCATCTACGCGGTGTACGTGCTGGTAGTTTAA

>TaEsi3-1-B_JZ888897.1

ATGGGCTCGGCAACAGTCCTGGAGGTGATCCTCGCCATCATCCTGCCTCCCGTCGGCGTCTTCCTGCGCTACAAACTCGGCGTGGAGTTCTGGATCTGTCTCTTGCTGACCATACTGGGGTACATACCGGGGATCATCTACGCGGTGTACGTGCTGGTAGTTTAA

>TaEsi3-1-D_JP881207.1

ATGGGCTCGGCAACAGTCCTGGAGGTGATCCTCGCCATCATCCTGCCTCCCGTCGGCGTCTTCCTGCGCTACAAACTCGGCGTGGAGTTCTGGATCTGTCTCTTGCTGACCATACTGGGGTACATACCGGGGATCATCTACGCGGTGTATGTGCTGGTAGTTTAA

>TaEsi3-2-A_HAAB01071723.1

ATGGCCTCAGCAACGTTCATAGAGGTGATCCTCGCCATCATCCTGCCTCCGGTCGGCGTCTTCCTGCGCTACGGCCTCGCCGTGGAGTTCTGGATCTGTCTCTTGCTGACCTTACTGGGGTACATACCGGGGATCATCTACGCGGTGTATGTGCTGGTGGCTTAA

>TaEsi3-2-D_HAAB01071724.1

ATGGCCTCAGCAACGTTCATAGAGGTGATCCTCGCCATCATCCTGCCTCCGGTCGGCGTCTTCCTGCGCTACGGCCTCGCCGTGGAGTTCTGGATCTGCCTCTTGCTGACCCTACTGGGGTACATACCGGGGATCATCTACGCGGTGTATGTGCTGGTGGCTTAA

>TaEsi3-3-A_CV767975.1

ATGGCGTCCCGAAGCTGCACCTTCGTCGAGATCCTGCTCGCCGTCATCCTGCCGCCGCTCGGCGTCTTCCTCCGCTACGGCTGCTGCAGCATGGAGTTCTTGATCTGTCTGCTGCTCACCATCCTGGGCTACATCCCCGGCATCATCTACGCCATCTACGTGCTCGTCGCGCATGGCTCGGCCTCGGAGGAGAGCGGCAAGGACTACGACGCCCTTGCTTGA

>TaEsi3-3-B_CJ925039.1

ATGGCGTCCCGGAGCTGCACCTTCGTGGAGATCCTGCTCGCCGTCATCCTGCCGCCGCTCGGCGTCTTCCTCCGCTACGGCTGCTGTAGCATGGAGTTCTTGATCTGCCTGCTGCTCACCATCCTGGGCTACATCCCCGGCATCATCTACGCCGTCTACGTGCTCGTCGCGCATGGCTCTGCCTCGGAGGAGAGCGGCAGGGACTACGACGCCCTTGGTTGA

>TaEsi3-3-D_CJ595273.1 CJ591008.1 CJ615800.1

ATGGCGTCCCGGAGCTGCACCTTCCTGGAGATCCTGCTCGCCGTCATCCTGCCGCCGCTCGGCGTCTTCCTCCGCTACGGCTGCTGCAGCATGGAGTTCTTGATCTGCCTGCTGCTCACCATCCTGGGCTACATCCCCGGCATCATCTACGCCGTCTACGTGCTCGTCGCGCATGGCTCCGCCTCGGAGGAGAGCGGCAGGGACTACGACGCCCTTGCTTGA

>TaEsi3-4-A_CA665474.1

ATGGCGGGCACGGCCAACTGCATCGACATCATCCTCGCCATCATCCTCCCGCCCCTCGGCGTCTTCCTCAAGTTCGGCTGCGGGCACGAGTTCTGGATCTGCCTCCTGCTCACCTTCCTCGGGTACATCCCGGGGATCATCTACGCCATCTACGCCATCACCAAGTAA

>TaEsi3-4-B_CJ901293.1

ATGGCGGGCACGGCCAACTGCATCGACATCATCCTCGCCATCATCCTCCCGCCCCTCGGCGTCTTCCTCAAGTTCGGCTGCGGGCACGAGTTCTGGATCTGCCTCCTGCTCACCTTCCTCGGGTACATCCCGGGGATCATCTACGCCATCTACGCCATCACCAAGTAA

>TaESI3-4-D_JV989019.1

ATGGCGGGCACGGCCAACTGCATCGACATCATCCTCGCCATCATCCTCCCGCCCCTCGGCGTCTTCCTCAAGTTCGGATGCGGGCACGAGTTCTGGATCTGCCTCTTGCTGACCTTCCTCGGGTACATCCCGGGTATCATCTACGCCATCTACGCCATCACCAAGTAA

>TaEsi3-5-A_HAAB01033003.1

ATGGCGGACGAGGGGACGGCCAACTGCATCGACATCATCCTCGCCATCATCCTGCCGCCGCTCGGCGTCTTCTTCAAGTTCGCCTGCGGGATCGAGTTCTGGATCTGCTTGCTGCTCACCTTCTTCGGCTACCTCCCCGGCATCATCTACGCCGTCTGGGTCATCACCAAGTAG

>TaEsi3-5-B_JV846264.1

ATGGCGGACGAGGGGACCGCCAACTGCATCGACATCATCCTCGCCATCATCCTGCCGCCGCTCGGCGTCTTCTTCAAGTTCGCCTGCGGGATCGAGTTCTGGATCTGCTTGCTGCTCACCTTCTTCGGCTACCTCCCCGGCATCATCTACGCCGTCTGGGTCATCACCAAGTAG

>TaEsi3-5-D_CJ854183.1

ATGGCGGACGAGGGGACGGCCAACTGCATCGACATCATCCTCGCCATCATCCTGCCGCCGCTCGGCGTCTTCTTCAAGTTCGCCTGCGGGATCGAGTTCTGGATCTGCTTGCTGCTCACCTTCTTCGGCTACCTCCCCGGCATCATCTACGCCGTCTGGGTCATCACCAAGTAG

>TaEsi3-6-A_CJ671046.1

ATGAGCTCCGGCGGCTGCTCGACGTGCCTGGAGATCATCTTCGCCGCCGTGCTCCCGCCGCTCGGCGTCTTCTTCCGGTACGGCTGGTGCAGCTCGGAGTTCTTCATCTCGCTGCCGCTGACGATACTCGGCTACGTCCCCGGCATCATCTACTCCGTCTACGTCATCCTGAAGACGCCGCCGGAGCTGCCGAGCATCGACGGCGAGCGGCCCTACTACATCCTCGCCTGA

>TaEsi3-6-B_CJ562290.1

ATGAGCTACTCCGGCGGCTGCTCGACGTGCCTGGAGATCGTCTTCGCCGCCGTGCTCCCGCCGCTCGGCGTCTTCTTCCGGTACGGCTGGTGCAGCTCGGAGTTCTTCATCTCGCTGCCGCTGACGATGCTCGGCTACGTCCCCGGCATCATCTACTCCGTCTACGTCATCCTGAAGACGCCGCCGGAGCTGCCGAGCATCGACGGCGAGCGGCCCTACTACATCCTCGCCTGA

>TaEsi3-6-D_CJ559253.1

ATGAGCTACTCCGGCGGCTGCTCGACGTGCCTGGAGATCGTCTTCGCCGCCGTGCTCCCGCCGCTCGGCGTCTTCTTCCGGTACGGCTGGTGCAGCTCGGAGTTCTTCATCTCGCTGCCGCTGACGATACTCGGCTACGTCCCCGGCATCATCTACTCCGTCTATGTCATCCTGAAGACGCCGCCGGAGCTGCCGAGCATCGACGGCGACCGGCCATACTACATCCTCGCCTAA

>TaEsi3-7-A_BJ261574.1

ATGGGGCTGTGCTCGTGCTGCTGCCGGTGCCTGGAGATCATGTGCGCCATCCTCCTCCCGCCCCTCGGCGTCTGCCTCCGCCACGGCTGCTGCTCCATGGAGTTCTGGATCAGCGTGCTGCTCACCATCCTCGGCTACCTCCCCGGCGTCCTCTACGCCGCCTACGTCATCTGCTCCGTCGACCCCGACCGCGTCCGCCGCCACGACGACGACTACATCTACGTCGCCTGA

>TaEsi3-7-B_CJ725702.1

ATGGGGCTGTGCTCGTGCTGCTGCCGGTGCCTGGAGATCATGTGCGCCATCCTCCTCCCGCCCCTCGGCGTCTGCCTCCGCCACGGCTGCTGCTCCATGGAGTTCTGGATCAGCGTGCTGCTCACCATCCTCGGCTACCTGCCGGGCGTCCTCTACGCCGCCTACGTCATCTGCTCCGTCGACCCCGACCGCGTCCGCCGCCGCGGCGACAGCGACGACGACTACATCTACGTCGCCTGA

>TaEsi3-7-D_CJ825516.1

ATGGGGCTGTGCTCGTGCTGCTGCCGGTGCCTGGAGATCCTGTGCGCCATCCTCCTCCCGCCCCTCGGCGTCTGCCTCCGCCACGGCTGCTGCTCCATGGAGTTCTGGATCAGCGTGCTGCTCACCATCCTCGGCTACCTCCCCGGCGTCCTCTACGCCGCCTACGTCATCTGCTCCGTCGACCCCGACCGCGTCCGCCGCCGCGACGACGACTACATCTACGTCGCCTGA

>TaEsi3-8-A_CD909025.1

ATGGGCTCGGAGACGTTCGTGGAGATCCTGCTGGCCATACTGCTGCCGCCGGTCGGCGTCTTCCTCCGCTACGGCATCGGCGTGGAGTTCTGGATATGCCTGCTGCTCACCGTGCTGGGCTACATCCCGGGCATCATCTACGCCATCTTCGTCCTCGTCGCTTAG

>TaEsi3-8-B_HAAB01084472.1

ATGGGTTCGGAGACGTTCGTGGAGATCCTGCTGGCCATCCTGCTGCCGCCGGTCGGCGTCTTCCTCCGCTACGGCATCGGCGTGGAGTTCTGGATCTGCCTGCTGCTCACGGTGCTGGGCTACATCCCCGGCATCATCTACGCCATCTTCGTCCTCGTCGCATAG

>TaEsi3-8-D_CJ648786.1

ATGGGTTCGGAGACGTTCGTGGAGATCCTGCTGGCCATCTTGCTGCCGCCGGTCGGCGTCTTCCTCCGCTACGGCATCGGCGTGGAGTTCTGGATCTGCCTGCTGCTCACGGTGCTGGGCTACATCCCCGGCATCATCTACGCCATCTTCGTCCTCGTCGCATAG

>TaEsi3-9-A_HAAB01083453.1

ATGGCAGAAACCGCAGCGATAGCACCACCACCACAACCAATGGCACCACCGCAACCAGTGGAGGAAAACGCAACGGCGGCACCGTCACAACCAATGGCACCACTGCAACCAATGGCGGAAAGCGCAACTGTCGTTGTGGTGGTGCCACCACCACCGCCAGACGGCACCACGACATTCCTCTGCCTCATCCTCGCCTTCTTCATCCCTCCCCTCGGCGTTTTCCTCAAGTACAAATGTGAGATTGAATTCTGGATCTGCCTCATCCTAACATTCTTGGCCTACGCGCCGGGCATCATCTACGCCGTCTGGGTGATCGTGAAGTAG

>TaEsi3-9-B_BJ243843

ATGGCAGAAAGCGCAGTGATAGCACCACCACTACAACCAATGACACCACCGCAACCAATGGCAGAAAACGCAACGGAGGCACCACCACAACCAATGGCACCACCGCAACAAATGGCGGAAAACGCAACGGCCGCACCACCACCACAACCAATGGCGGAAAACGCAACGGCGGCACCACCACCACAACCAATGGTGGAAAACGCAACGGTGGTTGTGGTGGTGCCACCACCACCGCCAGACAGCACCACAACATTCCTCTGCCTCATCCTCGCCTTCTTCCTCCCTCCCCTCGGCGTTTTCCTCAAGTACAAATGCGAGATTGAATTCTGGATCTGCCTCATCCTAACATTCTTGGCCTACGCGCCGGGCATCATCTACGCCGTCTGGGTGATCGTAAAGTAG

>TaEsi3-9-D_BJ243706.1

ATGGCAGAAAGCACAGCAATAGCACCACCACCACAACCAATGGCACCACCGCAACCAGTGGAGGAAAACGCAACGGCGGCACCGCCGCAACCAATGGCACCACCGCAACCAATGGCGGAAAACGCAACGGCGGCACCACCGCAACCAATGGCGGAAAACGCAACTGTCGTTGTGGTGGTGCCACCACCACCGCCAGACGGCACCACGACATTCCTCTGCCTCATCCTCGCCTTCTTCATCCCTCCCCTCGGCGTTTTCCTCAAGTACAAATGTGAGATTGAATTCTGGATCTGCCTCATCCTAACATTCTTGGCCTACGCGCCGGGCATCATCTACGCCGTCTGGGTGATCGTAAAGTAG

>TaEsi3-10-A_ HX161660.1 HX161634.1 CJ823027.1 CA645450.1

ATGGCGTCCCGGAGCTGCACCTTCCTCGAGATCCTGCTCGCCATCATCCTGCCGCCGCTCGGCGTCTTCCTCCACTACGGCTGCTGCAGCATGGAGTTCTGCATCTGCCTGCTGCTCACCATCCTGGGCTACATCCCCGGCATCATCTACGCTGTCTACATGCTCGTCGCGCTCGGCTCGGAGGAGCGTGATAGGGACTACAACACACTTGCTTAA

>TaEsi3-10-B_ CA611646.1

ATGGCGTCCCGGAGCTGCACCTTCCTGGAGATCCTGCTCGCCATCATCCTGCCGCCGCTCGGTGTCTTCCTCCACTACGGCTGCTGCAGCATGGAGTTCTGCATCTGCCTGCTGCTCACCATCCTGGGCTACATCCCCGGCATCATCTACGCGGTCTACGTGCTTGTCGCGCTCGGCTCGGAGGAGCGGGATCGGGACTACGACACCCTTGCTTAA

>TaEsi3-10-D_BJ278420.1

ATGGCGTCCCGGAGCTGCACCTTCCTCGAGATCCTGCTCGCCATCATCCTGCCGCCGCTCGGCGTCTTCCTCCACTACGGCTGCTGCAGCATGGAGTTCTGCATCTGCCTGTTGCTCACCATCCTGGGCTACATCCCCGGCATCATCTACGCGGTCTACGTGCTCGTTGCGCTCGGCTCAGAGGAGCGTGATCGGGACTACGACACCCTTGCTTAA
