# Supplementary material for: Characterization of the Esi3/RCI2/PMP3 gene family in the Triticeae
Source: BMC Genomics. 2018 Dec 11;19:898. doi: 10.1186/s12864-018-5311-8 (PMC6288971; doi:10.1186/s12864-018-5311-8)
Supplement: Supplementary file 8 — Figure S1. Tissue specific expression of the Esi3 genes in thirteen T. aestivum tissue types assayed by microarray. (PDF 298 kb) [file 12864_2018_5311_MOESM8_ESM.pdf]

A

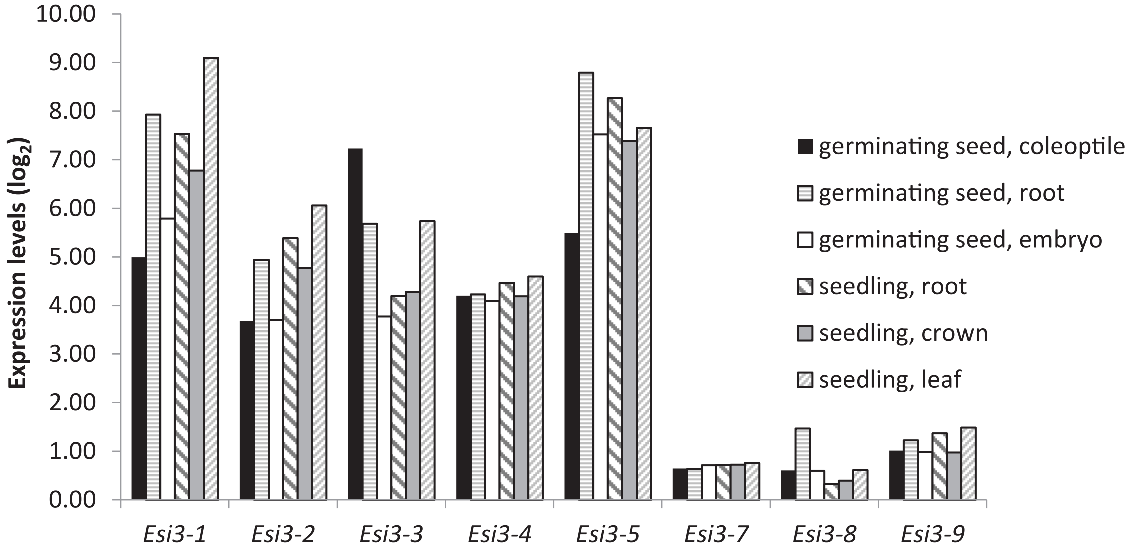

B

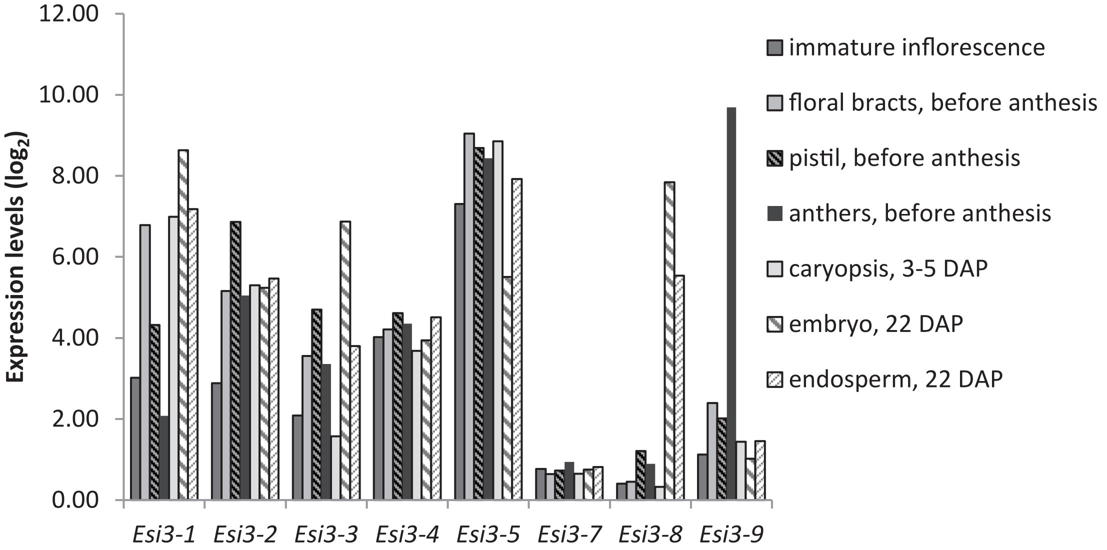

Additional Figure 1: Tissue specific expression of the *Esi3* genes across thirteen *T. aestivum* tissue types assayed by microarray. **(A)** Young tissue types ranging from germinating seed to seedling stages. **(B)** Mature tissue types before anthesis, 3-5 and 22 days after pollination (DAP). Values are RMA normalized and are in log_2 units. All microarray values had 4 subtracted, this was treated as background.
