# Supplementary material for: Characterization of the Esi3/RCI2/PMP3 gene family in the Triticeae
Source: BMC Genomics. 2018 Dec 11;19:898. doi: 10.1186/s12864-018-5311-8 (PMC6288971; doi:10.1186/s12864-018-5311-8)
Supplement: Supplementary file 15 — Figure S2. Molecular Phylogenetic analysis of the Esi3/RCI2/PMP3 protein sequences from nine species. (PDF 384 kb) [file 12864_2018_5311_MOESM15_ESM.pdf]

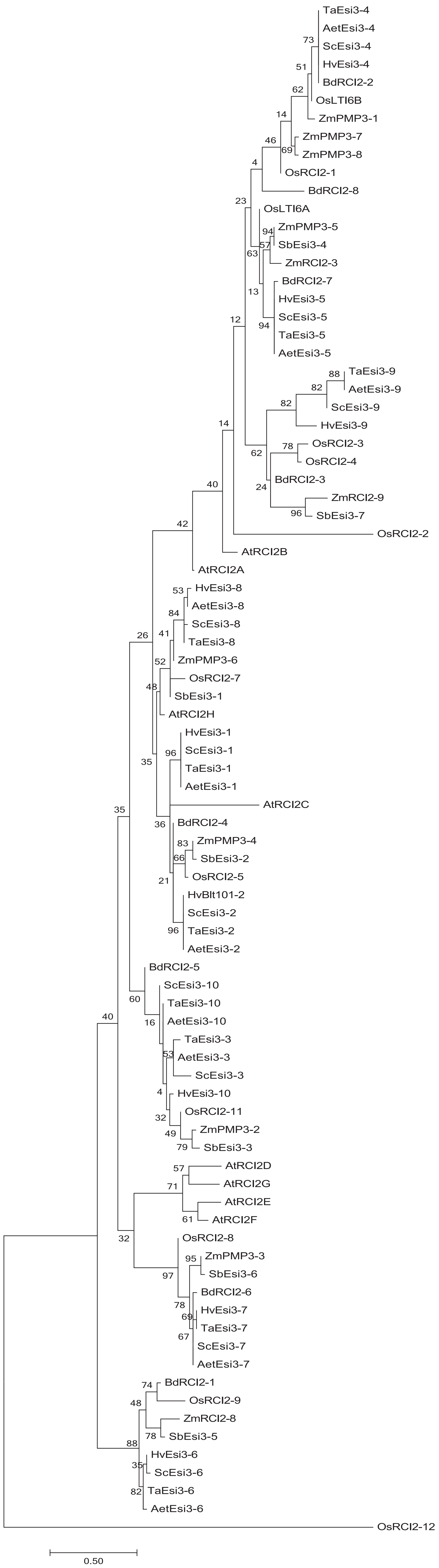

Additional Figure 2: Molecular Phylogenetic analysis of the Esi3/RCI2/PMP3 protein sequences from nine species. The evolutionary history was inferred by using the Maximum Likelihood method based on the Whelan And Goldman model [44]. The tree with the highest log likelihood (-2067.2128) is shown. The tree is drawn to scale, with branch lengths mea sured in the number of substitutions per site. The analysis involved 85 amino acid sequences. All positions containing gaps and missing data were eliminated. There were a total of 54 positions in the final dataset. The percentage of trees in which the associated taxa clustered together is shown next to the branches. The species abbreviatons are as follows; Ta- *Triticum aestivum*, Hv - *Hordeum vulgare*, At- *Arabidopsis thaliana*, Sb - *Sorghum bicolor*, Aet - *Aegilops tauschii*, Sc- *Secale cereale*, Zm- *Zea mays*, Os- *Oryza sativa*, Bd- *Brachypodium distachyon*.
