# Supplementary material for: Characterization of the Esi3/RCI2/PMP3 gene family in the Triticeae
Source: BMC Genomics. 2018 Dec 11;19:898. doi: 10.1186/s12864-018-5311-8 (PMC6288971; doi:10.1186/s12864-018-5311-8)
Supplement: Supplementary file 16 — Figure S3. Molecular phylogenetic analysis of the Esi3 genes from T. aestivum. (PDF 244 kb) [file 12864_2018_5311_MOESM16_ESM.pdf]

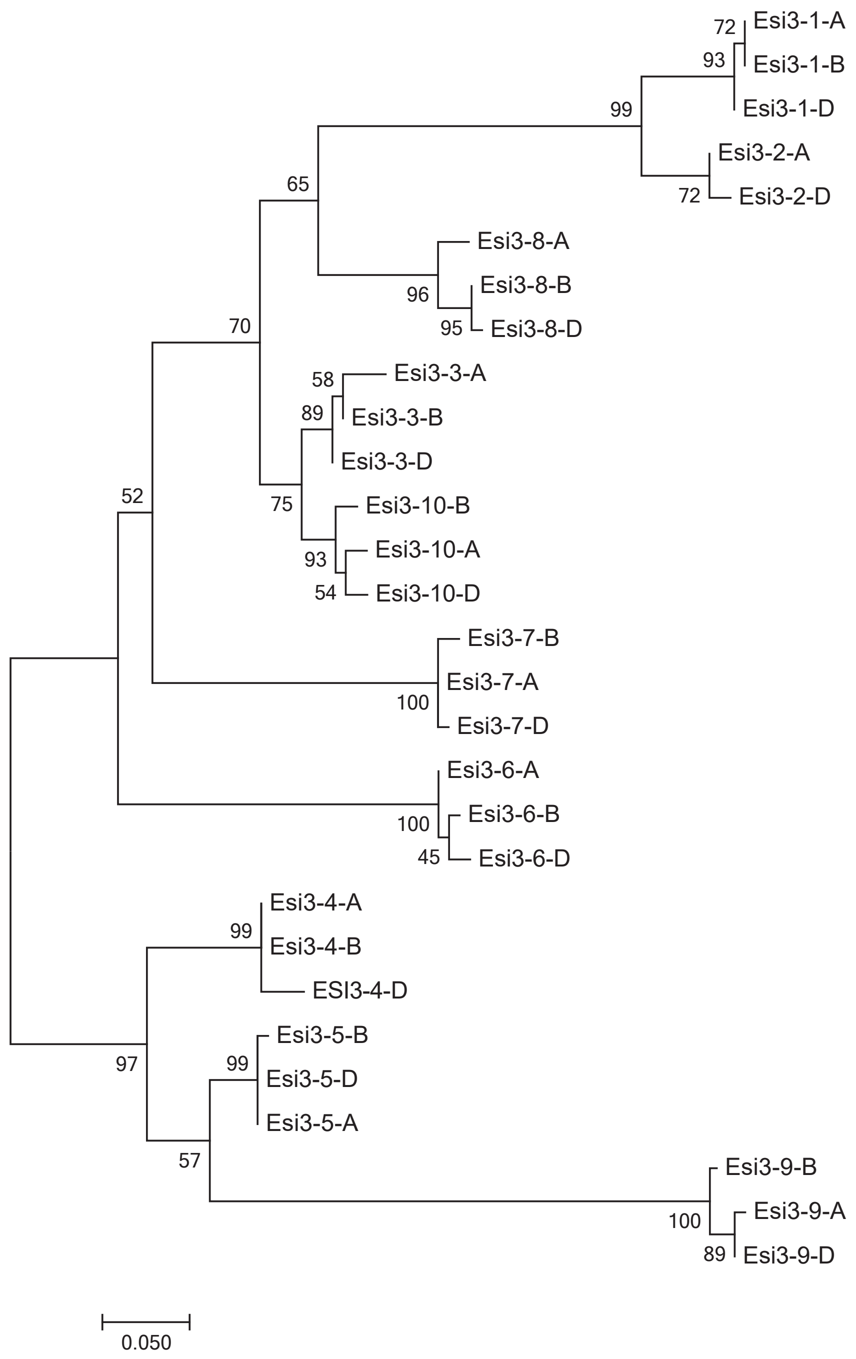

Additional Figure 3: Molecular phylogenetic analysis of the *Esi3* genes from *T. aestivum* by Maximum Likelihood method based on the Jukes-Cantor model [42]. The tree with the highest log likelihood (-1283.8239) is shown. The percentage of tress in which the associated taxa clustered together is shown next to the branches. A discrete Gamma distribution was used to model evolutionary rate differences among sites (5 categories (+G, parameter = 1.1637)). The tree is drawn to scale, with branch lengths measured in the number of sustitutions per site. The analysis involved 29 nucleotide sequences.
